# Transitions From Frailty States to Cardiovascular Events: An 11-Year Prospective Study of Community-Dwelling Older People

**DOI:** 10.1016/j.cjca.2025.12.002

**Published:** 2025-12-12

**Authors:** Aung Zaw Zaw Phyo, Andrew Tonkin, Sara E. Espinoza, Rory Wolfe, Suzanne G. Orchard, Robyn L. Woods, Joanne Ryan

**Affiliations:** a School of Public Health and Preventive Medicine, Monash University, Melbourne, Victoria, Australia; b Center for Translational Geroscience, Department of Medicine, Cedars-Sinai Medical Center, Los Angeles, California, USA

**Keywords:** Frailty transition, cardiovascular, sex, sociodemographic, older people

## Abstract

**Background:**

This study aims to quantify transition probabilities between frailty states (not-frail, pre-frail, frail) and from different frailty states to the occurrence of a cardiovascular disease (CVD) event; and to examine how age, sex and sociodemographic factors influence these transitions.

**Methods:**

18,077 initially healthy individuals (91% Caucasians, 56% females) aged ≥65 years enrolled in the ASPREE study who had no prior CVD event and ADL (Activity of Daily Living) disability at recruitment were followed for a median of 7.4 years. Frailty was annually assessed using a 64-item Frailty Index. Continuous-time multi-state Markov modelling was used.

**Results:**

The estimated transition probabilities from frail to CVD increased over time (11% at five years, and 18% at ten years) and consistently exceeded the corresponding five- and ten-year transition probabilities from pre-frail to CVD (8% and 14% respectively). Compared to males, females had a 26% higher relative risk of progressing to a pre-frail/frail state; however, they had about 50% lower relative risk of transitioning from pre-frail/frail to CVD. Older age, socioeconomic and geographic disparities were associated with up to 38% higher relative risk of worsening frailty and progression to CVD. Similar findings were observed when using the Fried phenotype.

**Conclusion:**

The probability of transiting from pre-frail/frail to CVD increased over time, even among initially healthy older people. Targeted prevention strategies may be helpful to delay frailty progression and reduce CVD risk in older age, particularly socioeconomically disadvantaged individuals or those residing outside major cities, and different approaches may be required in females and males.

## Central Illustration.

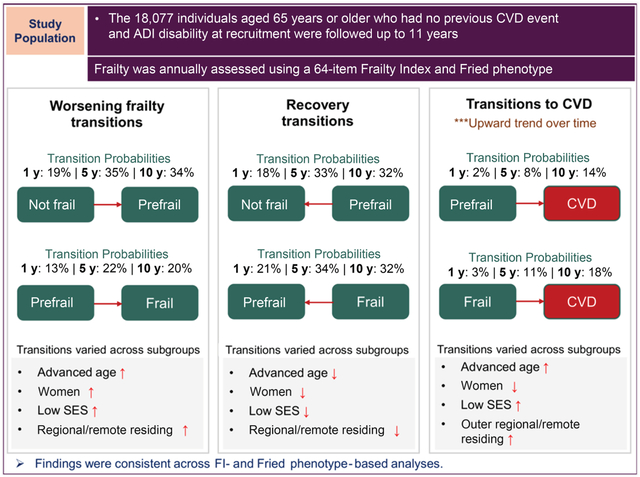

Transitions between frailty states and from frailty states to cardiovascular events among community-dwelling older people: influence of age, sex, socioeconomic status, and area of residence. ADL, activities of daily living; CVD, cardiovascular disease; SES, socioeconomic status.

## RÉSUMÉ

**Contexte:**

Cette étude visait à quantifier les probabilités de transition entre, d’une part, les différents états de fragilité (non fragile, pré-fragile, fragile), et, d’autre part, entre ceux-ci et l’occurrence d’une manifestation liée à une maladie cardiovasculaire (CV). Les auteurs ont aussi examiné de quelle façon l’âge, le sexe et les facteurs sociodémographiques influent sur ces transitions.

**Méthodologie:**

En tout, 18 077 personnes (91 % de race blanche, 56 % de femmes) âgées de 65 ans ou plus, en bonne santé au départ et sans antécédents de manifestations liées à une maladie CV ou d’incapacité dans les activités de la vie quotidienne (AVQ) au moment de leur sélection dans l’étude ASPREE, ont fait l’objet d’un suivi médian de 7,4 ans. Le degré de fragilité a été évalué annuellement à l’aide d’un indice de fragilité à 64 items et un modèle markovien à états multiples continu dans le temps a été utilisé.

**Résultats:**

La probabilité estimée de transition d’un état de fragilité à l’apparition d’une maladie CV augmente au fil du temps (de 11 % à 5 ans à 18 % à 10 ans) et a systématiquement dépassé la probabilité de transition correspondante d’un état pré-fragile à la maladie CV à 5 ans et à 10 ans (8 % et 14 %, respectivement). Les femmes présentaient un risque relatif de transition vers un état pré-fragile ou fragile 26 % plus élevé que chez les hommes; toutefois, leur risque relatif de transition d’un état pré-fragile/fragile vers la maladie CV était environ 50 % moins élevé. L’âge avancé et les disparités socio-économiques et géographiques étaient associés à une augmentation allant jusqu’à 38 % du risque relatif d’aggravation du degré de fragilité et de progression vers une maladie CV. Des résultats similaires ont été observés en utilisant les critères phénotypiques de Fried.

**Conclusion:**

La probabilité de transition d’un état pré-fragile/fragile vers la maladie CV augmente au fil du temps, même chez les personnes âgées en bonne santé au départ. Des stratégies de prévention ciblées pourraient permettre de retarder la progression de l’état de fragilité et de réduire le risque de maladie CV chez les personnes âgées, en particulier chez les personnes défavorisées sur le plan socio-économique ou celles qui résident hors des grands centres urbains. Des approches différentes pourraient en outre être requises chez les femmes et chez les hommes.

The growing burden of cardiovascular disease (CVD) in older adults represents a significant public health challenge.^1^ Frailty is a common, multifaceted geriatric syndrome characterized by cumulative pathological deficits in biological systems that increase susceptibility to stressors and illnesses.^2^ Previous studies revealed that prefrail or frail individuals at one time point (ie, baseline) had an increased risk of an incident CVD event over time.^3,4^ This finding suggests frailty is an early marker of increased cardiovascular vulnerabilities, positioning frailty as a target for interventions to reduce CVD risk.

Because frailty is a dynamic condition, some people transition from a prefrail state to a not frail state, whereas others progress to being frail.^2,5,6^ These different frailty transitions likely influence the risk of CVD differentially.^6^ However, traditional longitudinal studies often overlook the dynamic nature of frailty transitions by only examining frailty at a single static time point or the net change between 2 time points.^3,4,6^ This provides a limited understanding of how individuals transition between different frailty states over time, particularly in relation to the transition from different frailty states to developing CVD events among older people. Understanding these transition patterns can help enhance the detection of individuals most vulnerable to CVD. This, in turn, could aid in identifying critical points for intervention and inform health care planning to allocate resources where they are needed most.

The first aim of this study was to quantify transition probabilities between frailty states and from different frailty states to the occurrence of a CVD event among community-dwelling older adults. The second aim of this study was to assess how age, sex, socioeconomic status (SES) and area of residence influence these transitions between frailty states and to CVD events. On the basis of the social determinants of health framework, SES and residential context are crucial factors that influence lifestyle choices and access to health care, which in turn shape their overall health.^7–9^ Additionally, age and sex are well known nonmodifiable factors that contribute to differences in resilience to stressors, frailty burden, and CVD risk.^7,10,11^ Determining whether the transitions between frailty states and to CVD events vary across these subgroups will help identify individuals who would benefit most from targeted prevention strategies.

## Methods

### Study population

This study used longitudinal data from participants in the **ASP**irin in **R**educing **E**vents in the **E**lderly (ASPREE) study.^12^ ASPREE was a double-blinded, multicentre, randomized and placebo-controlled clinical trial conducted to test the effectiveness of daily low-dose aspirin in prolonging the primary end point of disability-free survival among older people when used in primary prevention.^12,13^ From 2010 through 2014, ASPREE recruited 19,114 initially healthy individuals (87% Australian) aged 70 years or older (65 years or older for US minorities) who had no previous CVD events, or other known 5-year life-limiting conditions as judged by their treating physician. Full details of the ASPREE study design and inclusion criteria have been presented elsewhere.^14^ The ASPREE cohort was followed prospectively with annual in-person assessments, and the clinical trial ended in June 2017. ASPREE showed no significant difference between the aspirin and placebo groups in disability-free survival,^13^ CVD events,^15^ and frailty.^16^ The post-ASPREE observational study (ASPREE-XT), an ongoing follow-up observational study, continued post-trial to follow these participants annually, and extensive clinical measures and phenotypic data were collected. The present study used the XT04 data set (which included 11 years of annual in-person assessment data from baseline to 2022).

The ASPREE study complied with the Declaration of Helsinki and was approved by multiple Institutional Review Boards in the United States and Australia (www.aspree.org). All participants signed written informed consent for participation. The present study was approved by the Monash University Human Research Ethics Committee (MHREC 42957).

### Frailty assessment

Frailty was assessed at annual follow-up visits (over a maximum of 11 years) using the deficit-accumulation Frailty Index (FI).^17^ The 64-item FI (Supplemental Table S1) was calculated on the basis of yearly measures collected in ASPREE, following the method described by Rockwood et al.^17,18^ (Details in Supplemental Appendix S1). Each item was scored, ranging from 0 (deficit absent) to 1 (deficit present). The FI score was estimated when at least 50 items were available and was calculated as the number of deficits noted for each person, divided by the total number of possible deficits. The score ranged from 0 to 1. Individuals were classified as not frail (≤ 0.10), prefrail (> 0.10 and ≤ 0.21), or frail (> 0.21).^16,19^

### CVD event

CVD was a prespecified composite secondary end point of ASPREE, consisting of coronary heart disease death, nonfatal myocardial infarction (MI), fatal or nonfatal stroke, or hospitalization for heart failure (HHF).^15^ Information related to CVD subtypes is presented in Supplemental Appendix S2. All incident CVD events were adjudicated by expert clinical panels (from Australia and the United States), masked to treatment allocation.^15^ Detailed information regarding the CVD events has been reported elsewhere.^15^

### Statistical analysis

**Figure 1.**
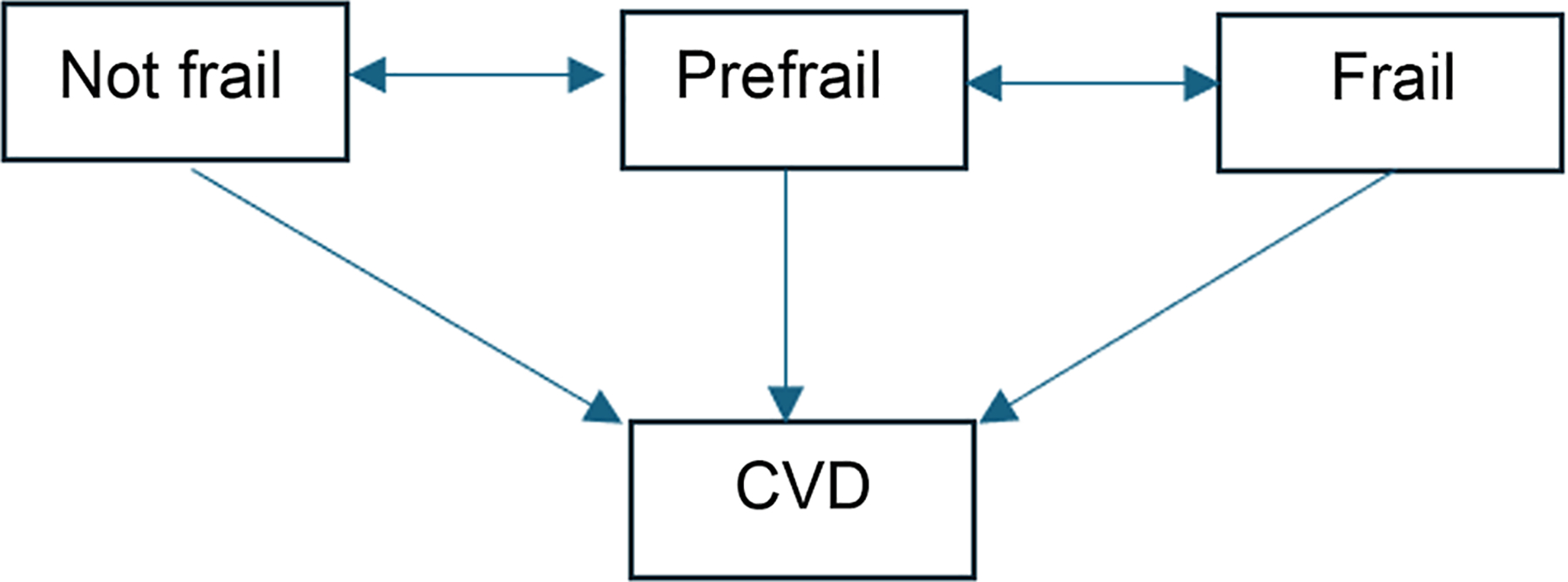
State transition diagram. CVD, cardiovascular disease.

Continuous-time multistate Markov (MSM) modelling was used in R (version 4.3.3; R Core Team, 2025, Vienna, Austria) with the R msm package version 1.7.1.^20^ The frailty state at each follow-up visit was treated as their current state, with incident CVD as the final absorbing state. Of note, because ASPREE assessed frailty only at each follow-up visit, exact frailty transition times were not available. We used the assumption that frailty transition intensities depend only on the current state (Markov assumption), and frailty transitions that occurred between follow-up visits were treated as interval-censored, as in the previous study.^21^ The exact date of the incident CVD diagnosis was used. The MSM model consisted of 4 states, comprising 3 frailty states: not frail, prefrail, and frail, and CVD as the absorbing state, with individuals transitioning between adjacent frailty states, as well as from any of the frailty states to CVD over time (Fig. 1).

First, an unadjusted MSM model was fitted to estimate mean sojourn times, representing the expected times in years spent in each state before transitioning to other states. Thereafter, the transition probabilities of being in each state at 1, 5, and 10 years were estimated, accounting for all possible transitions over the specific time, including indirect transitions through intermediate states (for example, from a not frail state to a frail state via a prefrail state). Next, age- and sex-adjusted MSM models were fitted to assess how age and sex influence the transitions between adjacent frailty states, as well as from any of the frailty states to CVD. Additionally, SES (low, middle, or high), or area of residence (major cities, inner regional, outer regional, or remote) were included separately in the age- and sex-adjusted MSM models to assess how SES or area of residence influences these transitions. SES was estimated using the Socio-Economic Indexes for Areas-Index of Relative Socioeconomic Advantage and Disadvantage for Australian participants,^22^ and the Social Deprivation Index for US participants.^23^ For Australian participants only, residential postcode information was available, which enabled the estimation of the area of residence. Moreover, because our cohort originated from a randomized clinical trial of aspirin,^12^ we further examined whether aspirin treatment influenced the transitions. As additional analyses, analyses were also repeated for the CVD subtypes—MI,^15^ stroke,^15^ and HHF.^15^

Moreover, sensitivity analyses were conducted by including deaths from non-CVD causes as a competing risk in the MSM (Supplemental Fig. S1). Stratified analyses were also undertaken to examine transition probabilities between frailty states and from different frailty states to the occurrence of a CVD event and to death from other causes according to age (younger than 74 years and 74 years or older, on the basis of the median), sex (male and female), SES (low, middle, and high), area of residence (major city, inner regional, outer regional, or remote), and treatment arms (placebo or aspirin).

Furthermore, to investigate the robustness of these findings, additional analyses were repeated using an alternative frailty assessment, the modified Fried phenotype.^24^ A modified Fried frailty phenotype (Fried) was defined on the basis of the 5 core criteria: (1) shrinking; (2) weakness; (3) exhaustion; (4) slowness; and (5) low activity.^19,24^ Full details of this Fried phenotype have been described previously.^18,19^ Each criterion was scored 0 for its absence or 1 for its presence. The total score ranged from 0 to 5. Individuals were classified as not frail (a score of 0), prefrail (a score of 1–2), or frail (a score of ≥ 3).

## Results

The present study included 18,077 community-dwelling older individuals who had information from at least 2 visits, enabling the transitions between states (ie, 2 frailty assessments or 1 frailty assessment and CVD as an absorbing state) to be examined (Table 1). Their median age was 73.9 years, and approximately half (56%) were female. Most (91%) were White Caucasian. Participants were evenly distributed across SES categories, with approximately one-quarter in the low group and a slightly higher proportion (approximately 38%) in the high group. Nearly half of the participants resided in regional or remote areas of Australia. Over a follow-up period of up to 11 years (median = 7.4 years; interquartile range, 5.1–8.7 years), 1719 (9.5%) of individuals experienced a CVD event.

### Transition probabilities between frailty states and to CVD

**Figure 2.**
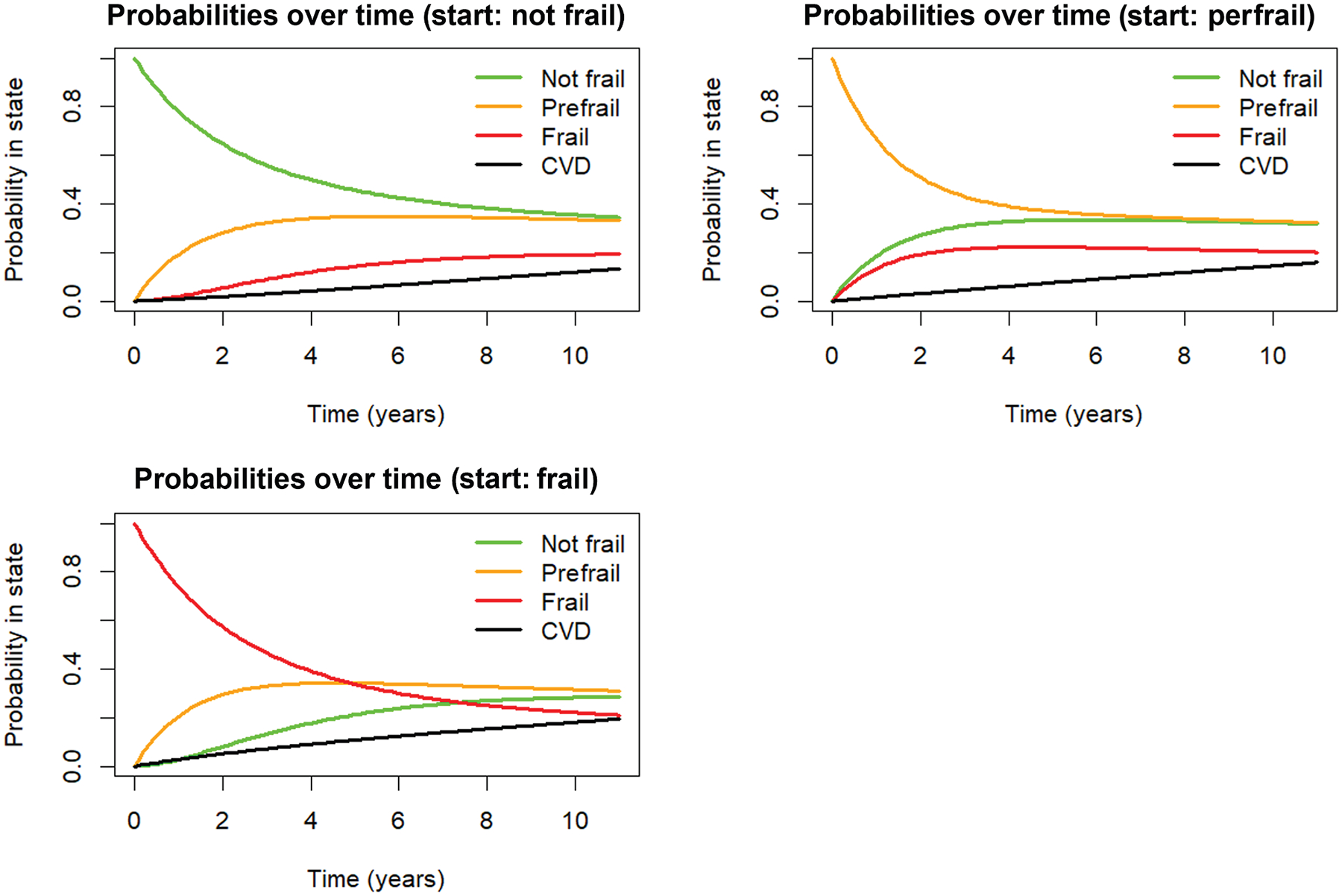
Probabilities of transitioning into different Frailty Index-defined frailty states and to a cardiovascular disease (CVD) event over time, starting from not frail, prefrail and frail.

Figure 2 shows a dynamic pattern of frailty state occupancy probabilities and the increasing risk of being in the absorbing CVD state over time. Probabilities of remaining in the starting state declined over time. Among individuals who started as prefrail, some transitioned back to a not frail state over time, and their likelihood of becoming frail or developing CVD increased gradually over time. Among individuals who started as frail, the likelihood of developing CVD increased progressively over time, with some transitioning back to prefrail or not frail.

**Figure 3.**
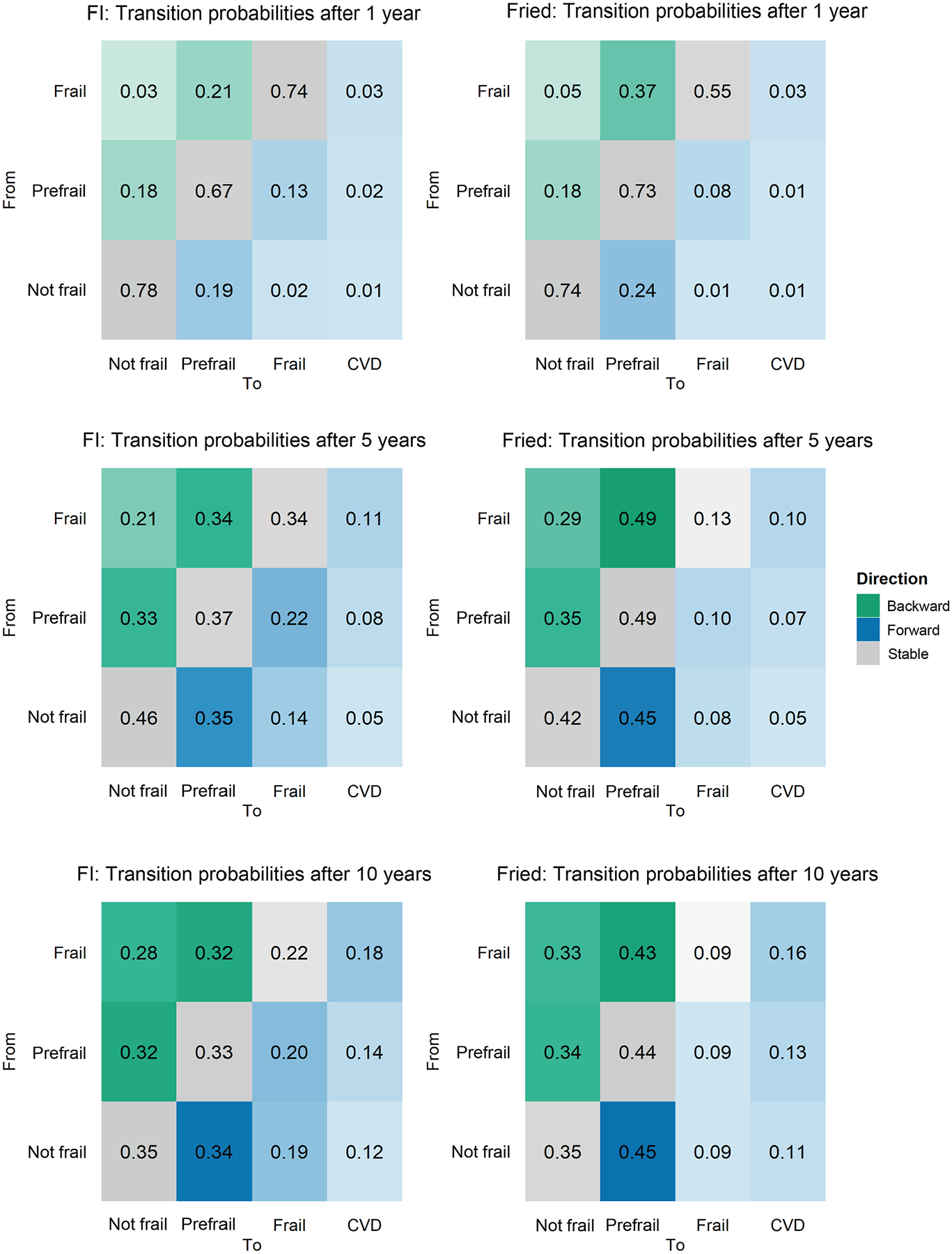
Transition probability between frailty states and from frailty state to a cardiovascular disease (CVD) event after 1 year, 5 years, and 10 years. FI, Frailty Index; Fried, Fried phenotype.

The estimated transition probabilities between frailty states and to a CVD event at 1, 5, and 10 years are presented in Figure 3. The participants’ mean time spent in the not frail, prefrail, and frail states before transitioning to another state was estimated to be 3.58 years (95% confidence interval [CI], 3.52–3.65), 2.12 years (95% CI, 2.09–2.16), and 3.04 years (95% CI, 2.95–3.13), respectively. The 1-year estimated transition probability from not frail to prefrail was 19%, whereas transitions from not frail to frail or CVD were relatively low (2% for frail and 1% for CVD). Over 5 years, the estimated transition probability from a not frail state to a frail state or developing a CVD event increased, with 14% becoming frail and 5% developing a CVD event. At 10 years, these rates increased to 19% for frailty and 12% for CVD (Fig. 3).

Notably, the estimated probability of forward transitioning from not frail to prefrail, or from prefrail to frail (worsening frailty status), was almost equal to that of backward transitioning from frail to prefrail, or from prefrail to not frail (improving frailty status). However, the chances of transitioning from prefrail to frail were always estimated to be lower (Fig. 3).

Individuals classified as prefrail were more likely to progress to frail or reverse to not frail rather than develop CVD. The 5-year estimated transition probabilities from prefrail to frail and to a CVD event were 22% and 8%, respectively. At 10 years, the estimated transition probabilities from prefrail to CVD increased to 14% (Central Illustration and Fig. 3).

The estimated transition probabilities from frail to CVD increased over time (3% at 1 year, 11% at 5 years, and 18% at 10 years) and consistently exceeded the corresponding transition probabilities from prefrail to CVD. The estimated transition probabilities from frail to prefrail were 21% at 1 year, increased to 34% at 5 years, and remained at 32% at 10 years (Central Illustration and Fig. 3).

The 1-year, 5-year, and 10-year transition probabilities from a frail state to all 3 CVD subtypes (MI, stroke, and HHF) were consistently highest, followed by prefrail, and lowest in not frail (Supplemental Table S2). Five- and 10-year transition probabilities from frail to stroke were the highest (ie, 5% at 5 years and 8% at 10 years) compared with MI (4% at 5 years and 7% at 10 years) and HHF (3% at 5 years and 4% at 10 years).

In additional analysis for Fried frailty transition modelling, the mean time spent in the not frail, prefrail, and frail states before transitioning to another state was estimated to be 2.91 years (95% CI, 2.84–2.99), 2.54 years (95% CI, 2.47–2.60), and 1.55 years (95% CI, 1.46–1.64), respectively. Transition probabilities between these frailty states and to CVD followed a pattern similar to those in the main analysis of the FI model across all time points (Fig. 3, Supplemental Fig. S2, Supplemental Table S2).

### Influence of age, sex, SES, and area of residence on transitions between frailty states and to CVD

A 1-year increase in chronological age was associated with an estimated 5% relative increase in risk of FI-defined frailty progression, a 7%−9% greater relative risk of developing CVD, depending on the originating frailty state, and a 3% lower relative risk of reverting to a less frail state (ie, prefrail to not frail, or frail to prefrail; Table 2). Compared with men, women had a 26% greater relative risk of progressing to a more severe frailty state (ie, not frail to prefrail or prefrail to frail) and were less likely to improve in frailty status. However, they had a 45%−49% lower relative risk of transition from a different frailty state to experiencing a CVD event.

Individuals with higher SES had up to 15% lower relative likelihood of frailty progression (ie, from not frail to prefrail, or from prefrail to frail) and were 11%−17% more likely to reverse to a less frail state (ie, from prefrail to not frail or from frail to prefrail) compared with those with lower SES. However, SES was not associated with the risk of transitioning to CVD. Individuals who resided in regional or remote areas were 6%−10% more likely to progress from a less frail state to a more severe frail state, whereas their likelihood of reversing to a less frail state (ie, prefrail to not frail) was 5% lower compared with those in metropolitan areas. The area of residence was not associated with risk of progressing from any frail state to CVD, except for individuals who lived in outer regional or remote areas, who had a 38% higher relative risk of moving from frail to a CVD event compared with those who lived in cities (Table 2). The treatment arm (aspirin vs placebo) was not associated with any transitions between frailty states or from any frail state to a CVD event (Table 2).

Similar associations were observed in the additional analyses of CVD subtypes (Supplemental Tables S3–S5). A 1-year increase in chronological age was consistently associated with a greater relative risk of transitioning from all frailty states to CVD subtypes (6% for MI, 7%−9% for stroke, and 12%−14% for HHF). Women consistently had lower relative risks of transitioning from a prefrail or frail state to each of the 3 CVD subtypes, with the largest reduction in relative risk observed for MI, where women had a 60% lower relative risk than men. Individuals with higher SES had a 29% lower relative risk of transitioning from prefrail to MI. Individuals who resided in inner regional areas had a 36% greater risk of transitioning from prefrail to MI than those in metropolitan areas. The area of residence was not associated with the risk of progressing from any frail state to stroke or HHF.

In the additional analyses using the Fried frailty phenotypes, the results were largely consistent with the findings of the FI (Table 2 and Supplemental Tables S3–S5). Women had a 13% greater relative likelihood of reversing from prefrail to not frail (Table 2). Individuals with high SES had a 40% lower relative risk of developing CVD from a frailty state compared with those with low SES. Individuals who resided in outer regional or remote areas had a 37% greater relative risk of transition from a prefrail state to CVD compared with those who lived in cities (Table 2).

### Sensitivity analysis

**Figure 4.**
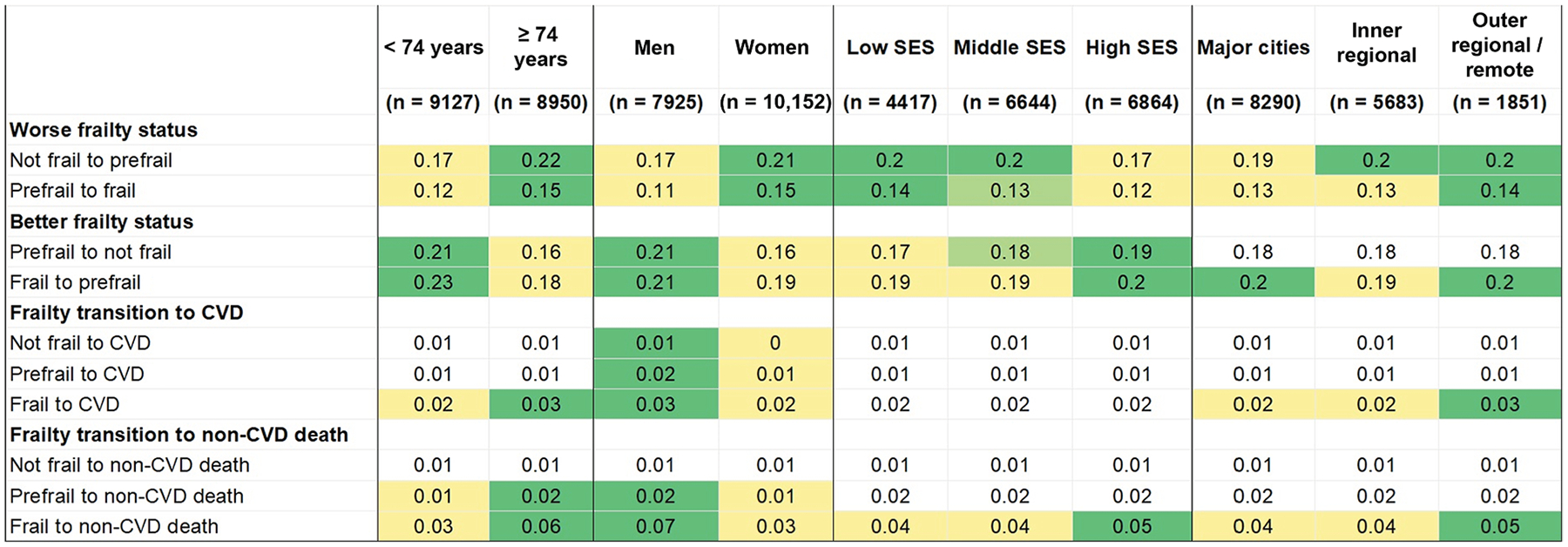
One-year transition probability matrix for the transitions between frailty states, from frailty states to a cardiovascular disease (CVD) event (absorbing state), and to death from non-CVD causes (competing risk), according to age group, sex, socioeconomic status (SES) and area of residence. Cells shaded in **green** indicate higher transition probabilities between comparison groups (eg, younger than 74 years vs 74 years or older, male vs female sex, SES strata, and area of residence strata) whereas **yellow** cells reflect comparatively lower probabilities between comparison groups. Unshaded cells represent transitions for which the probability was equal across comparison groups.

When death from non-CVD causes (2285 events) was included as a competing risk, the overall pattern of 1-year transition probabilities between frailty states and from frailty states to a CVD event remained similar to the main findings, but transition probabilities were slightly lower (Supplemental Tables S6 and S7). The 1-year probability of transiting from a prefrail or frail state to non-CVD death was 2% and 4%, respectively. Over a longer period, this transition probability to non-CVD death became noticeable (ie, 13%−22% at 5 years and 26%−35% at 10 years) whereas transition probabilities between frailty states were slightly lower compared with those of the main analysis. The 5- and 10-year transition probabilities from frailty states to a CVD event were attenuated when competing death from non-CVD causes was taken into account (Supplemental Tables S6 and S7). The findings from the stratified analyses (Fig. 4 and Supplemental Tables S8–S12) also showed that older age, female sex, low SES, and residents of outer regional or remote areas had higher transition probabilities to worse frailty and lower transition probabilities into frailty recovery. Those with advanced age, male sex, and residing in outer regional/remote areas had a slightly higher probability of transition from frail to CVD and non-CVD death after 1 year. For these groups, transition probabilities from prefrail or frail state to non-CVD death increased substantially after 5 years and 10 years.

## Discussion

To our knowledge, this is the first study to quantify the transition probability between different frailty states and transitioning from frailty states to incident CVD using a large community-based cohort of older people without previous CVD events. Our study bridges 2 major concerns within geriatric health: transitions to becoming frail and experiencing a CVD event. Our findings also show that although frailty progressed and transitioned to CVD over time, there was also potential for recovery. Notably, the likelihood of older individuals transitioning from a frail state back to a prefrail state over 5 years was comparable with that of remaining in a frail state, and greater than that of transitioning from a frail state to CVD. This result underscores the dynamic and reversible nature of frailty, and provides evidence that frailty is not an inevitably progressive condition. Further, our study provides new insights into how age, sex, and socioeconomic and geographic disparities influence dynamic frailty patterns and transition from frailty to CVD events, with important implications for health policy and prevention.

Previous studies provided evidence that prefrail and frail individuals at a single time point (ie, at the time of study entry) had a greater risk of incident CVD events over follow-up.^3,4^ Our present study extends the existing evidence by modelling dynamic transitions from different frailty states to CVD events over 1, 5, and 10 years, and offers a better understanding of how CVD risk accumulates over time. In this study, we observed that the likelihood of transitioning from prefrailty or frailty to an overall CVD event increased continuously over 10 years. This finding is consistent across CVD subtypes, with transition probabilities from frail to stroke or MI being slightly greater than for HHF, which reflects substantial cerebrovascular and cardiac vulnerability in the frail/prefrail group. Overall, our finding indicates that frailty assessment among older people could provide additional context for identifying those at greater CVD risk and inform intervention strategies to maintain functional health and prevent cardiovascular complications in later life.

Beyond the observed worsening of frailty over time, our study also illustrates the bidirectional, dynamic, and potentially reversible nature of frailty. This finding aligns with evidence from previous studies in which frailty transition was assessed as the net change between 2 time points over an average of 4–6 years.^25,26^ In our present modelling using the annual frailty status over up to 11 years with CVD as the absorbing state, individuals in the prefrail or frail state demonstrated transitions across all directions, toward recovery (ie, from prefrail to not frail), reversal to a less frail state (ie, from frail to prefrail), progression to frailty, and incident CVD. These findings, together with previous evidence,^25,26^ show that the prefrail and frail stages are critical windows for the timely identification and implementation of targeted strategies to mitigate the frailty burden, which, in turn, could help prevent adverse cardiovascular outcomes. In addition, we noticed that the reversed transition probabilities are slightly greater in the Fried phenotype than in FI. This finding might partly reflect how frailty is operationalized. For example, FI includes accumulated health deficits that cannot be reversed,^17^ whereas the Fried phenotype reflects functional performance, which might fluctuate with recovery or acute illness.^24,27^ Therefore, our observed transitions toward less severe frail states in this study could reflect improved resilience and well-being, as well as function and mobility related to recovery from illness, rather than a reversal of the underlying physiological process.

In this study older age influenced frailty progression and transitions from frailty to a subsequent CVD event, including CVD subtypes, even among initially “healthy” older people. This supports previous evidence that advancing age tends to accelerate transitions to greater frailty, accompanied by an age-dependent decline in biological and physiological resilience.^28^ Our current study reveals a previously overlooked aspect that, with increasing age, the chances of backward transition from frailty (ie, transition from frail to prefrail or prefrail to not frail) noticeably decrease, and frailty transitions tend to occur more in one direction, toward a greater frailty burden, ultimately associated with CVD events. This finding reflects the conceptualization of frailty as a multidimensional syndrome characterized by physiological decline in later life and highlights that a frailty continuum becomes progressively less reversible as people age.

Notably, when compared with men, women had a greater likelihood of frailty progression to more severe states; however, their risk of advancing from a prefrail or frail state to developing a CVD event was lower. The possible explanation could be a complex interaction of biological factors, sex hormones, social factors, and health care-seeking behaviours. For example, compared with men, women experience a faster decline in resting energy expenditure, which is associated with age-related loss of muscle mass and decreased metabolic rate of muscle tissue.^29^ This condition increases the likelihood of women becoming frail. In addition, our findings of sex differences in the risk of transitioning from a prefrail/frail state to developing CVD events align with well-established evidence that men have a greater risk of CVD than women.^30^ This disparity reflects that men are more likely to have high blood pressure, to smoke, and have central obesity, which are all atherosclerotic CVD risk factors^31^ and could also relate to underlying biological differences.^30,32,33^ For example, men have a decline in testosterone concentrations as they age, which is associated with a greater risk of fatal illnesses, including heart disease.^32,33^ Further, women often tend to have greater health care engagement and are naturally more likely to seek treatment for their illness,^34,35^ which together could lead to earlier detection and intervention and therefore buffer the effects of frailty on CVD risk. In contrast, even at similar frailty levels, men who perhaps under-report their symptoms and are more likely to delay seeking care,^35,36^ could perhaps experience a greater likelihood of progressing to worse health outcomes. This sex-associated divergence in frailty-CVD transition reflects the male-female health-survival paradox^37^ in which women live longer, and are more likely to experience disabilities, whereas men exhibit a greater risk of mortality from chronic health outcomes such as CVD.

Our findings also underscore the role of SES in shaping the dynamic progression of frailty and vulnerability. Evidence has shown that a high SES is associated with greater levels of health literacy, access to health care services, and better health outcomes.^38,39^ The current study extends this knowledge base by demonstrating that high SES not only influences the onset and progression of frailty but also the greater potential for frailty recovery in late life. This finding suggests that frailty recovery, which reflects improvement in physical functioning and broader aspects of health and resilience, might be possible for those with favourable SES, for whom there is a greater chance of getting timely and effective health care management for recovery from adverse health conditions. Moreover, our study showed that older individuals who reside in regional or remote areas (vs metropolitan) experience a greater risk of transition from a less frail state to a more severe frail state and from a frail state to CVD. This finding could reflect health inequalities in nonmetropolitan regions.^40^ For example, inadequate availability of allied health and social care supportive services, or limited access to local health services, necessitates travelling far for health care, resulting in delays in the detection and management of frailty and its complications, and a greater risk of adverse health outcomes, such as CVD, in already vulnerable individuals.^41^

### Clinical implications

Our findings from this large-scale research provide crucial data for health professionals and policymakers to better understand the transitions between different frailty states and the potential for developing CVD events from frailty states. This finding was consistent across CVD subtypes, with stroke and MI compared with HHF having slightly greater transition probabilities from frailty. These findings reinforce frailty’s role as an indicator of ageing-related vulnerability in the context of CVD, which indicates more attentive monitoring for CVD among prefrail/frail individuals. Furthermore, because we observed the consistent associations between advanced age and transitions from different frailty states to CVD, acknowledging frailty as part of the broader health profile of older individuals might help contextualize vulnerability to CVD in later life. Moreover, our age- and sex-stratified findings also show that older men, compared with their female counterparts, had a noticeably greater probability of transitioning from frailty states to CVD (Supplemental Table S13). This suggests that vulnerability might manifest differently in men and women, and frailty status could be an important consideration for older men in relation to CVD development. In addition, our findings on the influence of socioeconomic and geographic disparities on frailty progression and transitions from a frail state to CVD further call for targeted outreach and resource allocation in rural areas and for those who are socioeconomically disadvantaged. Together, these new insights from our study can help health care policymakers allocate resources cost-effectively and clinicians identify optimal targets for tailoring interventions to mitigate frailty, and provide knowledge for developing future strategies aimed at supporting healthy ageing, along with reducing CVD burden.

### Strengths and limitations

Our study has strengths and limitations. Using 11-year longitudinal data on clinical and phenotypic assessments, this study enabled the estimation of frailty at each available time point over time, using 2 of the most widely applied instruments: the 64-item FI, which captures multidimensional frailty, and the Fried phenotype, which represents physical frailty. Another strength was that this study was undertaken in a large cohort of community-dwelling older adults who had no previous CVD events or a known 5-year life-limiting condition at the time of enrollment. This cohort was followed over time for the occurrence of an incident CVD event, with rigourous ascertainment and adjudication by expert outcomes committees on the basis of laboratory test results and clinical records, rather than relying on administrative data sets. This enhances the quality of our study by ensuring accurate diagnosis of CVD events. Thus, the combination of a rich longitudinal data set with repeated frailty assessment over 11 years using 2 commonly used approaches—FI and Fried phenotype—and the rigourous adjudication of CVD events provided us with a unique opportunity to examine the estimated transition probabilities between frailty states and to CVD events among a large cohort of community-dwelling older people. This, in turn, provides a comprehensive and comparative perspective that yields new insights into the dynamic nature of frailty and its associated CVD risk. In addition, our ASPREE sample is diverse in terms of sex (56% female), with approximately 25% living in the most socioeconomically disadvantaged areas. Furthermore, Australian ASPREE participants were recruited across 5 states and territories, with approximately 48% living in regional and remote areas of Australia. They had standardized assessments conducted by centrally trained staff, ensuring that there was no differential ascertainment of data. Together, these factors minimize variation in data collection across staff and collection centres. Notably, our study measured frailty annually, which is a common practice in large-scale cohort studies. This might have overlooked some short-term transitions that occurred between our measurement waves, and the exact timing of transitions between frailty states was not available. This limitation might introduce some imprecision into our estimated results. Further, it is acknowledged that approximately 91% of our study participants identified themselves as White Caucasian. This could limit the generalizability of our findings to other racial and ethnic groups. Moreover, our study participants were free of CVD and activity of daily living disability at recruitment, which likely contributed to lower rates of frailty progression and greater chances of recovery than in a more clinically diverse patient setting. Of note, for the purpose of modelling, frailty (FI and Fried) was grouped into 3 states (not frail, prefrail, frail) using defined criteria that have been applied previously.^16,19,24,42^ It is acknowledged that FI is a continuous measure that reflects a graded spectrum of vulnerability; categorizing FI into 3 states is an analytical simplification required for the multistate model. Thus, transitions between FI-defined frailty states might represent relatively small changes in scores rather than marked biological shifts. Because we examined a large number of transitions that might increase the likelihood of chance findings, results should be interpreted with caution, with emphasis on the overall pattern of associations rather than individual estimates. Moreover, it should be acknowledged that when non-CVD deaths were incorporated into the main model as a competing risk, similar patterns were observed in short-term transition probabilities, but longer-term transition probabilities, particularly transitions from frailty to CVD, were attenuated.

## Conclusion

Our community-based study showed that the likelihood of transitioning from prefrailty or frailty to incident CVD increased continuously over time, even among initially healthy older people. With increasing age, the chances of recovery from frailty noticeably decrease, and frailty transitions tend to occur more in one direction, toward a greater frailty burden, ultimately leading to a greater risk of developing CVD. Our study also provides evidence that sex and socioeconomic and geographic disparities also influence the transition between frailty states and to CVD. Together, our study provides new insight into how frailty evolves over time and its transitions to CVD in later life, and offers important considerations for understanding ageing-related vulnerability (frailty) for CVD.

## Supplementary Material

Supplementary Material

To access the supplementary material accompanying this article, visit the online version of the *Canadian Journal of Cardiology* at www.onlinecjc.ca and at https://doi.org/10.1016/j.cjca.2025.12.002.

## Figures and Tables

**Table 1.** Baseline characteristics of the study participants according to the FI-defined frailty status at baseline

Characteristic	Total (N = 18,077)	Not frail (n = 8805)	Prefrail (n = 7627)	Frail (n = 1639)	*P* *
Age, years	73.9 (71.6–77.6)	73.3 (71.4–76.3)	74.5 (71.8–78.3)	75.7 (72.4–80.3)	**< 0.001**
Sex					
Male	7925 (43.8)	4515 (51.3)	2987 (39.2)	420 (25.6)	**< 0.001**
Female	10,152 (56.2)	4290 (48.7)	4640 (60.8)	1219 (74.4)	
Country					
Australia	15,871 (87.8)	7760 (88.1)	6710 (88.0)	1396 (85.2)	**0.003**
United States	2206 (12.2)	1045 (11.9)	917 (12.0)	243 (14.8)	
Ethno-racial group					
White Australian	15,540 (86.0)	7586 (86.2)	6581 (86.3)	1368 (83.5)	**< 0.001**
White American	1038 (5.7)	586 (6.7)	373 (4.9)	79 (4.8)	
Black/African American	791 (4.4)	294 (3.3)	374 (4.9)	122 (7.4)	
Hispanic/Latino/Asiatic/other	708 (3.9)	339 (3.9)	299 (3.9)	70 (4.3)	
Socioeconomic status					
Low	4417 (24.6)	1922 (22.0)	2022 (26.8)	472 (29.2)	**< 0.001**
Middle	6644 (37.1)	3167 (36.2)	2836 (37.5)	638 (39.5)	
High	6864 (38.3)	3656 (41.8)	2700 (35.7)	506 (31.3)	
Area of residence^†^					
Major cities of Australia	8290 (52.4)	4122 (53.3)	3442 (51.4)	723 (51.9)	0.07
Inner regional Australia	5683 (35.9)	2734 (35.4)	2425 (36.2)	522 (37.5)	
Outer regional or remote Australia	1851 (11.7)	879 (11.4)	824 (12.3)	148 (10.6)	
Frailty index score	0.10 (0.07–0.15)	0.07 (0.05–0.08)	0.14 (0.12–0.16)	0.25 (0.23–0.29)	**< 0.001**
Treatment					
Placebo	9091 (50.3)	4389 (49.9)	3895 (51.1)	802 (48.9)	0.15
Aspirin	8986 (49.7)	4416 (50.1)	3732 (48.9)	837 (51.1)	

Bold *P*-values represent a significant difference between groups at *P* < 0.05. Data are presented as n (%) or median (interquartile range), except where otherwise noted. Six participants had missing baseline frailty status data but were retained in the analysis because they provided frailty data from at least 2 subsequent follow-up visits, permitting estimation of their state transitions.

FI, Frailty Index.

* From χ^2^ or Kruskal-Wallis test.

† Australian participants only (approximately 88% of the total cohort).

**Table 2.** Influence of age, sex, SES, area of residence, and aspirin on transitions between frailty states and to a CVD event

	Worse frailty status		Better frailty status		Transition to CVD		
	Not frail to prefrail	Prefrail to frail	Prefrail to not frail	Frail to prefrail	Not frail to CVD	Prefrail to CVD	Frail to CVD
Transition between FI-defined frailty states and to CVD (N = 18,077)							
Ag_e_ (n = 18,077)	**1.05 (1.04–1.06)**	**1.04 (1.03–1.04)**	**0.97 (0.96–0.97)**	**0.97 (0.96–0.98)**	**1.07 (1.04–1.10)**	**1.07 (1.06–1.09)**	**1.09 (1.07–1.11)**
Sex							
Male (n = 7925)	Ref	Ref	Ref	Ref	Ref	Ref	Ref
Female (n = 10,152)	**1.26 (1.22–1.31)**	**1.26 (1.20–1.33)**	**0.79 (0.75–0.82)**	**0.82 (0.77–0.88)**	**0.52 (0.41–0.66)**	**0.51 (0.43–0.60)**	**0.55 (0.46–0.66)**
SES							
Low (n = 4417)	Ref	Ref	Ref	Ref	Ref	Ref	Ref
Middle (n = 6644)	0.99 (0.94–1.04)	**0.91 (0.85–0.96)**	**1.11 (1.05–1.17)**	1.02 (0.94–1.11)	0.77 (0.58–1.03)	0.97 (0.78–1,21)	0.98 (0.78–1.23)
High (n = 6864)	**0.85 (0.80–0.89)**	**0.85 (0.80–0.91)**	**1.17 (1.10–1.24)**	**1.11 (1.02–1.21)**	0.76 (0.57–1.00)	0.90 (0.72–1.13)	1.01 (0.80–1.27)
Area of residence							
Major cities (n = 8290)	Ref	Ref	Ref	Ref	Ref	Ref	Ref
Inner regional (n = 5683)	**1.06 (1.02–1.11)**	**1.07 (1.01–1.13)**	**0.95 (0.90–0.99)**	0.97 (0.90–1.04)	1.27 (0.99–1.62)	1.04 (0.86–1.26)	1.00 (0.81–1.23)
Outer regional/remote (n = 1851)	1.07 (1.00–1.14)	**1.10 (1.02–1.20)**	0.95 (0.88–1.02)	1.02 (0.91–1.14)	1.01 (0.68–1.52)	1.09 (0.82–1.45)	**1.38 (1.04–1.84)**
Treatment							
Placebo (n = 9091)	Ref	Ref	Ref	Ref	Ref	Ref	Ref
Aspirin (n = 8986)	0.98 (0.95–1.02)	1.02 (0.98–1.07)	1.00 (0.96–1.05)	1.00 (0.94–1.07)	1.07 (0.86–1.33)	0.91 (0.77–1.08)	1.07 (0.89–1.28)
Transition between Fried phenotype frailty states and to CVD (n = 15,920)							
Age (n = 15,920)	**1.03 (1.02–1.04)**	**1.08 (1.07–1.09)**	**0.93 (0.92–0.94)**	0.99 (0.98–1.00)	**1.07 (1.03–1.10)**	**1.08 (1.06–1.10)**	**1.08 (1.04–1.11)**
Sex							
Male (n = 7012)	Ref	Ref	Ref	Ref	Ref	Ref	Ref
Female (n = 8908)	**1.11 (1.06–1.17)**	**1.23 (1.11–1.36)**	**1.13 (1.06–1.21)**	1.01 (0.89–1.15)	**0.53 (0.41–0.67)**	**0.60 (0.50–0.73)**	**0.67 (0.48–0.93)**
SES							
Low (n = 3840)	Ref	Ref	Ref	Ref	Ref	Ref	Ref
Middle (n = 5870)	0.93 (0.87–1.00)	0.88 (0.78–1.00)	**1.18 (1.09–1.29)**	1.03 (0.88–1.20)	0.91 (0.67–1.24)	0.90 (0.71–1.15)	0.92 (0.63–1.34)
High (n = 6088)	0.95 (0.89–1.02)	0.87 (0.76–1.00)	**1.38 (1.27–1.50)**	**1.24 (1.06–1.45)**	0.82 (0.60–1.12)	0.95 (0.75–1.21)	**0.60 (0.39–0.94)**
Area of residence							
Major cities (n = 7377)	Ref	Ref	Ref	Ref	Ref	Ref	Ref
Inner regional (n = 5041)	1.01 (0.95–1.08)	**1.14 (1.01–1.28)**	0.99 (0.92–1.06)	0.94 (0.81–1.08)	1.08 (0.83–1.40)	1.09 (0.88–1.35)	1.06 (0.72–1.56)
Outer regional/remote (n = 1653)	**0.91 (0.83–0.99)**	1.17 (0.98–1.39)	0.98 (0.88–1.09)	0.93 (0.75–1.16)	0.84 (0.55–1.29)	**1.37 (1.01–1.85)**	1.35 (0.79–2.29)
Treatment							
Placebo (n = 8002)	Ref	Ref	Ref	Ref	Ref	Ref	Ref
Aspirin (n = 7918)	0.99 (0.94–1.05)	0.97 (0.88–1.08)	1.00 (0.93–1.06)	0.93 (0.82–1.05)	1.12 (0.89–1.42)	0.99 (0.82–1.19)	1.00 (0.72–1.38)

Data are presented as hazard ratio (95% confidence interval) for the listed transition, except where otherwise noted. Models were adjusted for age and sex. Bold represents a significant result at *P* < 0.05. CVD, cardiovascular disease; Ref, reference; SES, socioeconomic status.

## Data Availability

Data from the ASPREE study are available upon reasonable request to qualified researchers, subject to approval of the analyses by the principal investigators and a standard data-sharing agreement. Details regarding requests to access the data are available through the ASPREE Access Management Site (https://ams.aspree.org).
