# Supplementary material for: Transitions From Frailty States to Cardiovascular Events: An 11-Year Prospective Study of Community-Dwelling Older People
Source: Can J Cardiol. Author manuscript; Available in PMC 2026 May 24. (PMC13197884; doi:10.1016/j.cjca.2025.12.002)
Supplement: Supplementary Material [file NIHMS2175694-supplement-Supplementary_Material.docx]

**Supplemental materials**

**Supplemental Appendix ---------------------------------------------------------------- Page 2**

**Supplemental Tables -------------------------------------------------------------------- Page 3**

**Supplemental Figures ------------------------------------------------------------------- Page 23**

**Supplemental Appendix S1: Details of the 64-item Frailty Index**

The original frailty index (FI) of ASPREE comprised 67 items (Supplemental Table S1). In this study, we excluded the three items related to the CVD-related disease diagnoses, specifically stroke, myocardial infarction and hospitalisation for heart failure, from the FI calculation to avoid overlap.^1^ The 64 items include 8 disease diagnoses, such as osteoporosis, 13 disease indicators, such as central adiposity and smoking, 4 cognitive function measures, 2 physical performance measures (grip strength and gait speed), 11 mental and psychosocial deficits, and 26 functional deficits that relate to completing the activities of daily living. Full details describing the FI calculation have been reported previously.^1,2^

**Supplemental Appendix S2: CVD Subtypes**

Myocardial infarction was defined according to the joint guidelines of the European Society of Cardiology and the American College of Cardiology.^3^ Non-fatal stroke was identified based on the World Health Organization’s definition of rapidly developing clinical signs of focal or global disturbance of cerebral function lasting more than 24 h (unless interrupted by surgery or death), with no apparent cause other than ischaemic or haemorrhagic cerebrovascular disease.^3^ Hospitalization for heart failure was defined as any unplanned overnight stay or longer in a hospital or similar facility with heart failure as the principal reason for admission.^3^ Detailed information related to these specific CVD subtypes has been published previously.^3^

**References**

**1.** Phyo AZZ, Tonkin AM, Espinoza SE, et al. Frailty trajectories after a cardiovascular event among community-dwelling older people. Eur J Prev Cardiol. 2025: zwaf095.

**2.** Ryan J, Espinoza S, Ernst ME, et al. Validation of a deficit-accumulation frailty index in the ASPirin in Reducing Events in the Elderly Study and its predictive capacity for disability-free survival. J Gerontol A Biol Sci Med Sci. 2022;77(1):19-26.

**3.** McNeil JJ, Wolfe R, Woods RL, et al. Effect of Aspirin on cardiovascular events and bleeding in the healthy elderly. N Engl J Med. 2018;379(16):1509-18

**Supplemental Table S1. The 64-item Frailty Index ***

| **Disease diagnosis (8)** | **Source** |
| --- | --- |
| Cancer | ASPRRE Endpoint |
| Depression (CES-D-10 score) | ASPRRE Endpoint |
| Diabetes | Clinical measure & drugs |
| Gastroesophageal reflux disease | Self-report |
| Osteoarthritis | Self-report |
| Osteoporosis | Prescription drugs |
| Urinary incontinence | Prescription drugs |
| Major Hemorrhage (excluding stroke) | ASPREE Endpoint |
| **Disease indicators (13)** |  |
| Albuminuria | Clinical measure |
| Anemia | Clinical measure |
| Central adiposity | Clinical measure |
| Dyslipidemia | Clinical measure & drugs |
| Hospitalizations in last year | Medical report |
| Hypertension | Clinical measure |
| Hypotension | Clinical measure |
| Number of Drug Prescriptions | Prescription drugs |
| Obesity | Clinical measure |
| Chronic Kidney Disease | Clinical measure |
| Self-rated health status | SF-12 questionnaire |
| Smoking | Self-report |
| Underweight | Clinical measure |
| **Functional status (26)** |  |
| Difficulty with bathing | ADL questionnaire |
| Difficulty with dressing | ADL questionnaire |
| Difficulty with eating | ADL questionnaire |
| Difficulty with toileting | ADL questionnaire |
| Difficulty with transferring | ADL questionnaire |
| Difficulty walking | ADL questionnaire |
| Accomplished less | SF-12 questionnaire |
| Climbing stairs limited | SF-12 questionnaire |
| Limited in kind of work/activities | SF-12 questionnaire |
| Moderate activities limited | SF-12 questionnaire |
| Pain interfere with work | SF-12 questionnaire |
| Requires a walking aid | LIFE questions |
| Any walking outside | LIFE questions |
| Difficulty walking several blocks | FAST questionnaire |
| Difficulty walking 8–12 blocks | LIFE questions |
| Difficulty climbing 1 flight of stairs | FAST questionnaire |
| Difficulty getting into a car | FAST questionnaire |
| Difficulty gripping objects | FAST questionnaire |
| Difficulty lifting 10 pounds | FAST questionnaire |
| Difficulty lifting heavy objects | FAST questionnaire |
| Difficulty raising arms above head | FAST questionnaire |
| Difficulty running errands | FAST questionnaire |
| Difficulty walking 1 block | FAST questionnaire |
| Difficulty with light housework | FAST questionnaire |
| Difficulty with meal preparation | FAST questionnaire |
| Difficulty with shopping | LIFE questions |
| **Mental & Psychosocial (11)** |  |
| Accomplished less due to emotional problems | SF-12 questionnaire |
| A lot of energy | SF-12 questionnaire |
| Calm or peaceful | SF-12 questionnaire |
| Difficulty participating in community activities | FAST questionnaire |
| Difficulty taking care of family — FAST questionnaire | FAST questionnaire |
| Difficulty using the telephone | FAST questionnaire |
| Difficulty with financial management | FAST questionnaire |
| Difficulty visiting relatives/friends | FAST questionnaire |
| Downhearted and depressed | SF-12 questionnaire |
| Less careful in work/activities | SF-12 questionnaire |
| Social activities impacted by health | SF-12 questionnaire |
| **Cognition & Performance (6)** |  |
| Episodic memory, delayed recall | HVLT-R questionnaire |
| Low Global cognition | 3MS questionnaire |
| Low Psychomotor Speed | SDMT questionnaire |
| Low Language and Executive Function | COWAT questionnaire |
| Slow Gait speed over 3 meters | Clinical measure |
| Weak handgrip strength | Clinical measure |

Notes: 3MS: Modified Mini-Mental State Examination; ADL: Activity of Daily Living; CES-D: Center for the Epidemiologic Studies Depression scale; COWAT: Controlled Oral Word Association Test; HVLT-R: Hopkins Verbal Learning Test Revised; SBP: Systolic Blood Pressure; SDMT: Symbol Digit Modalities Test; SF-12: 12-item short-form questionnaire.

*The full details of the ASPREE frailty index have been published previously.

Ryan J, et. al. Validation of a Deficit-Accumulation Frailty Index in the ASPirin in Reducing Events in the Elderly Study and Its Predictive Capacity for Disability-Free Survival. J Gerontol A Biol Sci Med Sci, 2022, Vol. 77, No. 1, 19–26. Doi. https://doi.org/10.1093/gerona/glab225

**Supplemental Table S2**. Transition probability from frailty state to CVD subtypes - MI, Stroke and HHF after one year, five years and ten years

| **Frailty state From\To** | **MI** | **Stroke** | **HHF** |
| --- | --- | --- | --- |
| **Frailty index** | | | |
| **Transition probabilities after one year** | | | |
| **Frail** | 0.01 | 0.01 | 0.01 |
| **Pre Frail** | 0.01 | 0.01 | 0.00 |
| **Not Frail** | 0.00 | 0.00 | 0.00 |
|  | | | |
| **Transition probabilities after five years** | | | |
| **Frail** | 0.04 | 0.05 | 0.03 |
| **Pre Frail** | 0.03 | 0.03 | 0.01 |
| **Not Frail** | 0.02 | 0.02 | 0.01 |
|  | | | |
| **Transition probabilities after ten years** | | | |
| **Frail** | 0.07 | 0.08 | 0.04 |
| **Pre Frail** | 0.06 | 0.06 | 0.03 |
| **Not Frail** | 0.05 | 0.05 | 0.02 |
|  | | | |
| **Fried phenotype** | | | |
| **Transition probabilities after one year** |  |  |  |
| **Frail** | 0.01 | 0.01 | 0.01 |
| **Pre Frail** | 0.01 | 0.01 | 0.00 |
| **Not Frail** | 0.00 | 0.00 | 0.00 |
|  | | | |
| **Transition probabilities after five years** | | | |
| **Frail** | 0.03 | 0.04 | 0.02 |
| **Pre Frail** | 0.03 | 0.03 | 0.01 |
| **Not Frail** | 0.02 | 0.02 | 0.01 |
|  | | | |
| **Transition probabilities after ten years** | | | |
| **Frail** | 0.06 | 0.07 | 0.04 |
| **Pre Frail** | 0.05 | 0.06 | 0.02 |
| **Not Frail** | 0.05 | 0.05 | 0.02 |

Notes: MI, Myocardial Infarction, HHF, Hospitalization for Heart Failure

**Supplemental Table S3**. Influence of age, sex, SES, area of residence and aspirin on transitions from frailty states and to a myocardial infarction (MI) event

|  | **Hazard ratio (95% CI) for the listed transition** | | | | | | |
| --- | --- | --- | --- | --- | --- | --- | --- |
|  | **FI-defined frailty (N = 16969)** | | |  | **Fried phenotype frailty (N = 15087)** | | |
|  | **Not frail to MI** | **Pre-frail to MI** | **Frail to MI** |  | **Not frail to MI** | **Pre-frail to MI** | **Frail to MI** |
| **Age** | **1.06 (1.02, 1.11)** | **1.06 (1.03, 1.09)** | **1.06 (1.03, 1.09)** |  | 1.05 (1.00, 1.10) | **1.08 (1.04, 1.11)** | 1.06 (1.00, 1.12) |
| **Sex** |  | | |  |  | | |
| Males | Ref | Ref | Ref |  | Ref | Ref | Ref |
| Females | **0.23 (0.14, 0.37)** | **0.43 (0.33, 0.57)** | **0.40 (0.29, 0.56)** |  | **0.32 (0.22, 0.48)** | **0.52 (0.38, 0.72)** | 0.60 (0.34, 1.07) |
| **SES** |  | | |  |  | | |
| Low | Ref | Ref | Ref |  | Ref | Ref | Ref |
| Middle | 1.13 (0.69, 1.85) | 0.84 (0.61, 1.15) | 1.35 (0.85, 2.13) |  | 1.11 (0.72, 1.73) | 1.03 (0.68, 1.56) | 1.08 (0.54, 2.13) |
| High | 0.96 (0.59, 1.55) | **0.71 (0.51, 0.99)** | 1.47 (0.93, 2.34) |  | 0.74 (0.46, 1.18) | 1.04 (0.69, 1.56) | 0.72 (0.33, 1.58) |
| **Area of residence** |  | | |  |  | | |
| Major cities | Ref | Ref | Ref |  | Ref | Ref | Ref |
| Inner regional | 1.15 (0.78, 1.71) | **1.36 (1.02, 1.80)** | 0.77 (0.52, 1.15) |  | 1.12 (0.78, 1.60) | 1.17 (0.82, 1.67) | 1.06 (0.54, 2.05) |
| Outer regional/remote | 0.86 (0.44, 1.70) | 1.13 (0.72, 1.80) | 1.32 (0.79, 2.20) |  | 0.66 (0.34, 1.30) | **1.69 (1.06, 2.69)** | 1.19 (0.43, 3.25) |
| **Treatment** |  | | |  |  | | |
| Placebo | Ref | Ref | Ref |  | Ref | Ref | Ref |
| Aspirin | 0.95 (0.67, 1.40) | 0.97 (0.75, 1.30) | 1.26 (0.89, 1.80) |  | 1.17 (0.83, 1.60) | 1.03 (0.76, 1.40) | 0.89 (0.50, 1.60) |

Note: 611 MI events in FI-defined frailty analysis; 486 MI events in Fried phenotype frailty analysis

Models were adjusted for age and sex. Bold represents a significant result at p <0.05.

**Supplemental Table S4**. Influence of age, sex, SES, area of residence and aspirin on transitions from frailty states and to stroke event

|  | **Hazard ratio (95% CI) for the listed transition** | | | | | | |
| --- | --- | --- | --- | --- | --- | --- | --- |
|  | **FI-defined frailty (N = 17067)** | | |  | **Fried phenotype frailty (N = 15149)** | | |
|  | **Not frail to Stroke** | **Pre-frail to Stroke** | **Frail to Stroke** |  | **Not frail to Stroke** | **Pre-frail to Stroke** | **Frail to Stroke** |
| **Age** | **1.07 (1.02, 1.11)** | **1.09 (1.06, 1.11)** | **1.09 (1.07, 1.12)** |  | **1.08 (1.03, 1.13)** | **1.09 (1.06, 1.12)** | **1.07 (1.02, 1.12)** |
| **Sex** |  | | |  |  | | |
| Males | Ref | Ref | Ref |  | Ref | Ref | Ref |
| Females | 0.80 (0.58, 1.11) | **0.57 (0.43, 0.74)** | **0.72 (0.53, 0.99)** |  | 0.92 (0.64, 1.32) | **0.60 (0.45, 0.79)** | 0.66 (0.38, 1.13) |
| **SES** |  | | |  |  | | |
| Low | Ref | Ref | Ref |  | Ref | Ref | Ref |
| Middle | **0.64 (0.42, 0.97)** | 1.03 (0.72, 1.47) | 0.91 (0.62, 1.32) |  | 0.74 (0.45, 1.20) | 0.80 (0.55, 1.17) | 0.78 (0.42, 1.45) |
| High | 0.71 (0.48, 1.05) | 1.14 (0.81, 1.61) | 1.06 (0.73, 1.53) |  | 0.95 (0.60, 1.49) | 0.95 (0.67, 1.36) | 0.58 (0.29, 1.19) |
| **Area of residence** |  | | |  |  | | |
| Major cities | Ref | Ref | Ref |  | Ref | Ref | Ref |
| Inner regional | 1.31 (0.91, 1.89) | 0.77 (0.57, 1.06) | 1.13 (0.81, 1.59) |  | 0.94 (0.61, 1.43) | 1.01 (0.73, 1.39) | 1.20 (0.64, 2.25) |
| Outer regional/remote | 1.08 (0.61, 1.94) | 1.05 (0.69, 1.61) | 1.41 (0.87, 2.29) |  | 1.03 (0.57, 1.87) | 1.24 (0.78, 1.98) | 1.41 (0.59, 3.39) |
| **Treatment** |  | | |  |  | | |
| Placebo | Ref | Ref | Ref |  | Ref | Ref | Ref |
| Aspirin | 1.16 (0.84, 1.60) | 0.87 (0.67, 1.14) | 1.22 (0.91, 1.64) |  | 1.02 (0.71, 1.47) | 1.05 (0.79, 1.39) | 1.24 (0.72, 2.14) |

Note: 709 Stroke events in FI-defined frailty analysis; 548 Stroke events in Fried phenotype frailty analysis

Models were adjusted for age and sex. Bold represents a significant result at p <0.05.

**Supplemental Table S5**. Influence of age, sex, SES, area of residence and aspirin on transitions from frailty states and to Hospitalization for heart failure (HHF) event

|  | **Hazard ratio (95% CI) for the listed transition** | | | | | | |
| --- | --- | --- | --- | --- | --- | --- | --- |
|  | **FI-defined frailty (N = 16646)** | | |  | **Fried phenotype frailty (N = 14805)** | | |
|  | **Not frail to HHF** | **Pre-frail to HHF** | **Frail to HHF** |  | **Not frail to HHF** | **Pre-frail to HHF** | **Frail to HHF** |
| **Age** | **1.12 (1.03, 1.22)** | **1.14 (1.09, 1.19)** | **1.12 (1.09, 1.16)** |  | 1.09 (0.97, 1.23) | **1.14 (1.09, 1.19)** | **1.11 (1.05, 1.17)** |
| **Sex** |  | | |  |  | | |
| Males | Ref | Ref | Ref |  | Ref | Ref | Ref |
| Females | 0.98 (0.48, 2.00) | **0.55 (0.35, 0.89)** | **0.46 (0.32, 0.67)** |  | 0.61 (0.26, 1.44) | 0.92 (0.56, 1.50) | 0.56 (0.30, 1.04) |
| **SES** |  | | |  |  | | |
| Low | Ref | Ref | Ref |  | Ref | Ref | Ref |
| Middle | 0.73 (0.27, 1.97) | 1.92 (0.91, 4.05) | 1.04 (0.65, 1.66) |  | 1.46 (0.33, 6.50) | 1.28 (0.63, 2.58) | 1.39 (0.61, 3.13) |
| High | 0.86 (0.35, 2.12) | 1.36 (0.62, 2.97) | 1.00 (0.62, 1.62) |  | 1.41 (0.33, 6.10) | 1.13 (0.56, 2.30) | 0.92 (0.38, 2.25) |
| **Area of residence** |  | | |  |  | | |
| Major cities | Ref | Ref | Ref |  | Ref | Ref | Ref |
| Inner regional | 2.10 (1.00,4.42) | 1.14 (0.66,1.98) | 0.90 (0.59,1.38) |  | 2.58 (0.96, 6.93) | 0.93 (0.54, 1.58) | 0.55 (0.24, 1.28) |
| Outer regional/remote | 0.82 (0.16,4.20) | 0.98 (0.40,2.38) | 1.16 (0.63,2.13) |  | 0.58 (0.05, 7.30) | 0.86 (0.36, 2.05) | 0.92 (0.33, 2.57) |
| **Treatment** |  | | |  |  | | |
| Placebo | Ref | Ref | Ref |  | Ref | Ref | Ref |
| Aspirin | 1.32 (0.64, 2.72) | 0.84 (0.52, 1.35) | 0.89 (0.62, 1.29) |  | 1.45 (0.61, 3.47) | 0.96 (0.59, 1.58) | 0.67 (0.35, 1.27) |

Note: 288 HHF events in FI-defined frailty analysis; 204 HHF events in Fried phenotype frailty analysis

Models were adjusted for age and sex. Bold represents a significant result at p <0.05.

**Supplemental Table S6.** Sensitivity analyses: Transition probabilities between FI-defined frailty states and from FI-defined frailty state to a CVD event (absorbing state) and death from non-CVD causes (competing risk) after one year, five years and ten years

| **Frailty state From\To** | **Not Frail** | **Pre-Frail** | **Frail** | **CVD** | **Death from non-CVD causes** |
| --- | --- | --- | --- | --- | --- |
| **Transition probabilities after one year** |  |  |  |  |  |
| **Frail** | 0.03 | 0.20 | 0.71 | 0.02 | 0.04 |
| **Pre Frail** | 0.18 | 0.66 | 0.13 | 0.01 | 0.02 |
| **Not Frail** | 0.78 | 0.19 | 0.02 | 0.01 | 0.01 |
|  |  |  |  |  |  |
| **Transition probabilities after five years** |  |  |  |  |  |
| **Frail** | 0.19 | 0.29 | 0.28 | 0.01 | 0.22 |
| **Pre Frail** | 0.31 | 0.34 | 0.20 | 0.01 | 0.13 |
| **Not Frail** | 0.44 | 0.33 | 0.13 | 0.01 | 0.09 |
|  |  |  |  |  |  |
| **Transition probabilities after ten years** |  |  |  |  |  |
| **Frail** | 0.23 | 0.25 | 0.16 | 0.01 | 0.35 |
| **Pre Frail** | 0.28 | 0.28 | 0.17 | 0.01 | 0.26 |
| **Not Frail** | 0.32 | 0.30 | 0.16 | 0.01 | 0.21 |
|  |  |  |  |  |  |

The mean time spent in no frail before moving to another state was 3.51 years (95%CI: 3.45 to 3.58)

The mean time spent in pre-frail before moving to another state was 2.07 years (95%CI: 2.04 to 2.10)

The mean time spent in frail before moving to another state was 2.71 years (95%CI: 2.64 to 2.79)

**Supplemental Table S7.** Sensitivity analyses: Transition probabilities between Fried phenotype frailty states and from Fried phenotype frailty state to a CVD event (absorbing state) and death from non-CVD causes (competing risk) after one year, five years and ten years

| **Frailty state From\To** | **Not Frail** | **Pre-Frail** | **Frail** | **CVD** | **Death from non-CVD causes** |
| --- | --- | --- | --- | --- | --- |
| **Transition probabilities after one year** |  |  |  |  |  |
| **Frail** | 0.05 | 0.35 | 0.52 | 0.03 | 0.04 |
| **Pre Frail** | 0.18 | 0.72 | 0.07 | 0.01 | 0.01 |
| **Not Frail** | 0.74 | 0.24 | 0.01 | 0.01 | 0.01 |
|  |  |  |  |  |  |
| **Transition probabilities after five years** |  |  |  |  |  |
| **Frail** | 0.26 | 0.42 | 0.11 | 0.02 | 0.20 |
| **Pre Frail** | 0.33 | 0.45 | 0.09 | 0.01 | 0.11 |
| **Not Frail** | 0.41 | 0.43 | 0.07 | 0.01 | 0.08 |
|  |  |  |  |  |  |
| **Transition probabilities after ten years** |  |  |  |  |  |
| **Frail** | 0.27 | 0.35 | 0.07 | 0.01 | 0.30 |
| **Pre Frail** | 0.31 | 0.39 | 0.07 | 0.01 | 0.22 |
| **Not Frail** | 0.33 | 0.40 | 0.07 | 0.01 | 0.19 |
|  |  |  |  |  |  |

The mean time spent in no frail before moving to another state was 2.89 years (95%CI: 2.82 to 2.96)

The mean time spent in pre-frail before moving to another state was 2.48 years (95%CI: 2.41 to 2.54)

The mean time spent in frail before moving to another state was 1.46 years (95%CI: 1.38 to 1.54)

**Supplemental Table S8.** Transition probability matrix between frailty states and to a CVD event (absorbing state), and to death from non-CVD causes (competing risk) in the <74 years and ≥74 years age group separately

|  | **FI** | | **Fried** | |
| --- | --- | --- | --- | --- |
|  | <74 years  (n = 9127) | ≥74 years  (n = 8950) | <74 years  (n = 8256) | ≥74 years  (n = 7664) |
| **1-year** |  |  |  |  |
| **Worse frailty status** |  |  |  |  |
| Not-frail to pre-frail | 0.17 | 0.22 | 0.22 | 0.26 |
| Pre-frail to frail | 0.12 | 0.15 | 0.05 | 0.09 |
| **Better frailty status** |  |  |  |  |
| Pre-frail to not-frail | 0.21 | 0.16 | 0.24 | 0.13 |
| Frail to pre-frail | 0.23 | 0.18 | 0.41 | 0.34 |
| **Frailty transition to CVD** |  |  |  |  |
| Not-frail to CVD | 0.01 | 0.01 | 0.01 | 0.01 |
| Pre-frail to CVD | 0.01 | 0.01 | 0.01 | 0.02 |
| Frail to CVD | 0.02 | 0.03 | 0.02 | 0.03 |
| **Frailty transition to non-CVD death** |  |  |  |  |
| Not-frail to non-CVD death | 0.01 | 0.01 | 0.00 | 0.01 |
| Pre-frail to non-CVD death | 0.01 | 0.02 | 0.01 | 0.02 |
| Frail to non-CVD death | 0.03 | 0.06 | 0.03 | 0.05 |
| **5-year** |  |  |  |  |
| **Worse frailty status** |  |  |  |  |
| Not-frail to pre-frail | 0.32 | 0.35 | 0.41 | 0.47 |
| Pre-frail to frail | 0.17 | 0.23 | 0.06 | 0.12 |
| **Better frailty status** |  |  |  |  |
| Pre-frail to not-frail | 0.39 | 0.25 | 0.43 | 0.24 |
| Frail to pre-frail | 0.33 | 0.27 | 0.43 | 0.43 |
| **Frailty transition to CVD** |  |  |  |  |
| Not-frail to CVD | 0.01 | 0.01 | 0.01 | 0.02 |
| Pre-frail to CVD | 0.01 | 0.01 | 0.01 | 0.02 |
| Frail to CVD | 0.01 | 0.02 | 0.01 | 0.02 |
| **Frailty transition to non-CVD death** |  |  |  |  |
| Not-frail to non-CVD death | 0.06 | 0.12 | 0.06 | 0.10 |
| Pre-frail to non-CVD death | 0.09 | 0.18 | 0.07 | 0.15 |
| Frail to non-CVD death | 0.15 | 0.28 | 0.12 | 0.24 |
| **10-year** |  |  |  |  |
| **Worse frailty status** |  |  |  |  |
| Not-frail to pre-frail | 0.30 | 0.29 | 0.39 | 0.41 |
| Pre-frail to frail | 0.14 | 0.18 | 0.05 | 0.09 |
| **Better frailty status** |  |  |  |  |
| Pre-frail to not-frail | 0.37 | 0.20 | 0.41 | 0.21 |
| Frail to pre-frail | 0.28 | 0.22 | 0.36 | 0.34 |
| **Frailty transition to CVD** |  |  |  |  |
| Not-frail to CVD | 0.01 | 0.01 | 0.01 | 0.02 |
| Pre-frail to CVD | 0.01 | 0.01 | 0.01 | 0.01 |
| Frail to CVD | 0.01 | 0.01 | 0.01 | 0.01 |
| **Frailty transition to non-CVD death** |  |  |  |  |
| Not-frail to non-CVD death | 0.14 | 0.28 | 0.13 | 0.24 |
| Pre-frail to non-CVD death | 0.18 | 0.34 | 0.15 | 0.29 |
| Frail to non-CVD death | 0.24 | 0.44 | 0.20 | 0.38 |

**Supplemental Table S9.** Transition probability matrix between frailty states and to a CVD event (absorbing state), and to death from non-CVD causes (competing risk) in males and females separately

|  | **FI** | | **Fried** | |
| --- | --- | --- | --- | --- |
|  | Males  (n = 7925) | Females  (n = 10152) | Males  (n = 7012) | Females  (n = 8908) |
| **1-year** |  |  |  |  |
| **Worse frailty status** |  |  |  |  |
| Not-frail to pre-frail | 0.17 | 0.21 | 0.23 | 0.25 |
| Pre-frail to frail | 0.11 | 0.15 | 0.07 | 0.08 |
| **Better frailty status** |  |  |  |  |
| Pre-frail to not-frail | 0.21 | 0.16 | 0.17 | 0.19 |
| Frail to pre-frail | 0.21 | 0.19 | 0.35 | 0.35 |
| **Frailty transition to CVD** |  |  |  |  |
| Not-frail to CVD | 0.01 | 0.00 | 0.01 | 0.01 |
| Pre-frail to CVD | 0.02 | 0.01 | 0.02 | 0.01 |
| Frail to CVD | 0.03 | 0.02 | 0.04 | 0.03 |
| **Frailty transition to non-CVD death** |  |  |  |  |
| Not-frail to non-CVD death | 0.01 | 0.01 | 0.01 | 0.00 |
| Pre-frail to non-CVD death | 0.02 | 0.01 | 0.02 | 0.01 |
| Frail to non-CVD death | 0.07 | 0.03 | 0.06 | 0.04 |
| **5-year** |  |  |  |  |
| **Worse frailty status** |  |  |  |  |
| Not-frail to pre-frail | 0.30 | 0.36 | 0.42 | 0.44 |
| Pre-frail to frail | 0.14 | 0.24 | 0.08 | 0.10 |
| **Better frailty status** |  |  |  |  |
| Pre-frail to not-frail | 0.36 | 0.28 | 0.32 | 0.34 |
| Frail to pre-frail | 0.27 | 0.31 | 0.41 | 0.43 |
| **Frailty transition to CVD** |  |  |  |  |
| Not-frail to CVD | 0.01 | 0.01 | 0.02 | 0.01 |
| Pre-frail to CVD | 0.01 | 0.01 | 0.02 | 0.01 |
| Frail to CVD | 0.02 | 0.01 | 0.02 | 0.01 |
| **Frailty transition to non-CVD death** |  |  |  |  |
| Not-frail to non-CVD death | 0.11 | 0.07 | 0.09 | 0.06 |
| Pre-frail to non-CVD death | 0.18 | 0.11 | 0.14 | 0.09 |
| Frail to non-CVD death | 0.30 | 0.18 | 0.24 | 0.17 |
| **10-year** |  |  |  |  |
| **Worse frailty status** |  |  |  |  |
| Not-frail to pre-frail | 0.26 | 0.33 | 0.38 | 0.42 |
| Pre-frail to frail | 0.10 | 0.21 | 0.06 | 0.08 |
| **Better frailty status** |  |  |  |  |
| Pre-frail to not-frail | 0.32 | 0.25 | 0.29 | 0.32 |
| Frail to pre-frail | 0.20 | 0.28 | 0.32 | 0.37 |
| **Frailty transition to CVD** |  |  |  |  |
| Not-frail to CVD | 0.01 | 0.01 | 0.02 | 0.01 |
| Pre-frail to CVD | 0.01 | 0.01 | 0.01 | 0.01 |
| Frail to CVD | 0.01 | 0.01 | 0.01 | 0.01 |
| **Frailty transition to non-CVD death** |  |  |  |  |
| Not-frail to non-CVD death | 0.25 | 0.17 | 0.22 | 0.15 |
| Pre-frail to non-CVD death | 0.32 | 0.22 | 0.27 | 0.19 |
| Frail to non-CVD death | 0.45 | 0.30 | 0.36 | 0.26 |

**Supplemental Table S10.** Transition probability matrix between frailty states and to a CVD event (absorbing state), and to death from non-CVD causes (competing risk) in the placebo and aspirin groups separately

|  | **FI** | | **Fried** | |
| --- | --- | --- | --- | --- |
|  | Placebo  (n = 9091) | Aspirin  (n = 8986) | Placebo  (n = 8002) | Aspirin  (n = 7918) |
| **1-year** |  |  |  |  |
| **Worse frailty status** |  |  |  |  |
| Not-frail to pre-frail | 0.19 | 0.19 | 0.24 | 0.24 |
| Pre-frail to frail | 0.13 | 0.13 | 0.07 | 0.07 |
| **Better frailty status** |  |  |  |  |
| Pre-frail to not-frail | 0.18 | 0.18 | 0.18 | 0.18 |
| Frail to pre-frail | 0.20 | 0.19 | 0.36 | 0.34 |
| **Frailty transition to CVD** |  |  |  |  |
| Not-frail to CVD | 0.01 | 0.01 | 0.01 | 0.01 |
| Pre-frail to CVD | 0.01 | 0.01 | 0.01 | 0.01 |
| Frail to CVD | 0.02 | 0.02 | 0.03 | 0.03 |
| **Frailty transition to non-CVD death** |  |  |  |  |
| Not-frail to non-CVD death | 0.01 | 0.01 | 0.01 | 0.01 |
| Pre-frail to non-CVD death | 0.02 | 0.02 | 0.01 | 0.01 |
| Frail to non-CVD death | 0.04 | 0.05 | 0.04 | 0.04 |
| **5-year** |  |  |  |  |
| **Worse frailty status** |  |  |  |  |
| Not-frail to pre-frail | 0.33 | 0.33 | 0.43 | 0.43 |
| Pre-frail to frail | 0.20 | 0.20 | 0.09 | 0.09 |
| **Better frailty status** |  |  |  |  |
| Pre-frail to not-frail | 0.32 | 0.31 | 0.33 | 0.33 |
| Frail to pre-frail | 0.30 | 0.29 | 0.42 | 0.42 |
| **Frailty transition to CVD** |  |  |  |  |
| Not-frail to CVD | 0.01 | 0.01 | 0.01 | 0.01 |
| Pre-frail to CVD | 0.01 | 0.01 | 0.01 | 0.02 |
| Frail to CVD | 0.01 | 0.01 | 0.02 | 0.02 |
| **Frailty transition to non-CVD death** |  |  |  |  |
| Not-frail to non-CVD death | 0.09 | 0.09 | 0.08 | 0.08 |
| Pre-frail to non-CVD death | 0.13 | 0.14 | 0.11 | 0.12 |
| Frail to non-CVD death | 0.22 | 0.23 | 0.20 | 0.20 |
| **10-year** |  |  |  |  |
| **Worse frailty status** |  |  |  |  |
| Not-frail to pre-frail | 0.30 | 0.29 | 0.40 | 0.40 |
| Pre-frail to frail | 0.17 | 0.16 | 0.07 | 0.07 |
| **Better frailty status** |  |  |  |  |
| Pre-frail to not-frail | 0.28 | 0.28 | 0.31 | 0.30 |
| Frail to pre-frail | 0.25 | 0.24 | 0.35 | 0.34 |
| **Frailty transition to CVD** |  |  |  |  |
| Not-frail to CVD | 0.01 | 0.01 | 0.01 | 0.01 |
| Pre-frail to CVD | 0.01 | 0.01 | 0.01 | 0.01 |
| Frail to CVD | 0.01 | 0.01 | 0.01 | 0.01 |
| **Frailty transition to non-CVD death** |  |  |  |  |
| Not-frail to non-CVD death | 0.21 | 0.21 | 0.18 | 0.19 |
| Pre-frail to non-CVD death | 0.26 | 0.27 | 0.22 | 0.23 |
| Frail to non-CVD death | 0.35 | 0.36 | 0.30 | 0.31 |

**Supplemental Table S11.** Transition probability matrix between frailty states and to a CVD event (absorbing state), and to death from non-CVD causes (competing risk), according to low, middle and high SES status separately

|  | **FI** | | | **Fried** | |  |
| --- | --- | --- | --- | --- | --- | --- |
|  | Low SES  (n = 4417) | Middle SES  (n = 6644) | High SES  (n = 6864) | Low SES  (n = 3840) | Middle SES  (n = 5870) | High SES  (n = 6088) |
| **1-year** |  |  |  |  |  |  |
| **Worse frailty status** |  |  |  |  |  |  |
| Not-frail to pre-frail | 0.20 | 0.20 | 0.17 | 0.25 | 0.24 | 0.23 |
| Pre-frail to frail | 0.14 | 0.13 | 0.12 | 0.08 | 0.07 | 0.07 |
| **Better frailty status** |  |  |  |  |  |  |
| Pre-frail to not-frail | 0.17 | 0.18 | 0.19 | 0.16 | 0.18 | 0.20 |
| Frail to pre-frail | 0.19 | 0.19 | 0.20 | 0.35 | 0.34 | 0.37 |
| **Frailty transition to CVD** |  |  |  |  |  |  |
| Not-frail to CVD | 0.01 | 0.01 | 0.01 | 0.01 | 0.01 | 0.01 |
| Pre-frail to CVD | 0.01 | 0.01 | 0.01 | 0.01 | 0.01 | 0.01 |
| Frail to CVD | 0.02 | 0.02 | 0.02 | 0.03 | 0.03 | 0.03 |
| **Frailty transition to non-CVD death** |  |  |  |  |  |  |
| Not-frail to non-CVD death | 0.01 | 0.01 | 0.01 | 0.01 | 0.01 | 0.01 |
| Pre-frail to non-CVD death | 0.02 | 0.02 | 0.02 | 0.01 | 0.01 | 0.01 |
| Frail to non-CVD death | 0.04 | 0.04 | 0.05 | 0.05 | 0.05 | 0.04 |
| **5-year** |  |  |  |  |  |  |
| **Worse frailty status** |  |  |  |  |  |  |
| Not-frail to pre-frail | 0.34 | 0.34 | 0.32 | 0.45 | 0.43 | 0.42 |
| Pre-frail to frail | 0.22 | 0.20 | 0.18 | 0.10 | 0.09 | 0.08 |
| **Better frailty status** |  |  |  |  |  |  |
| Pre-frail to not-frail | 0.28 | 0.31 | 0.35 | 0.29 | 0.32 | 0.36 |
| Frail to pre-frail | 0.30 | 0.30 | 0.29 | 0.43 | 0.42 | 0.42 |
| **Frailty transition to CVD** |  |  |  |  |  |  |
| Not-frail to CVD | 0.01 | 0.01 | 0.01 | 0.02 | 0.01 | 0.01 |
| Pre-frail to CVD | 0.01 | 0.01 | 0.01 | 0.02 | 0.02 | 0.01 |
| Frail to CVD | 0.01 | 0.01 | 0.01 | 0.02 | 0.02 | 0.01 |
| **Frailty transition to non-CVD death** |  |  |  |  |  |  |
| Not-frail to non-CVD death | 0.10 | 0.09 | 0.08 | 0.08 | 0.08 | 0.07 |
| Pre-frail to non-CVD death | 0.14 | 0.13 | 0.13 | 0.12 | 0.11 | 0.11 |
| Frail to non-CVD death | 0.23 | 0.21 | 0.23 | 0.22 | 0.21 | 0.18 |
| **10-year** |  |  |  |  |  |  |
| **Worse frailty status** |  |  |  |  |  |  |
| Not-frail to pre-frail | 0.30 | 0.30 | 0.29 | 0.41 | 0.40 | 0.39 |
| Pre-frail to frail | 0.18 | 0.17 | 0.14 | 0.08 | 0.07 | 0.06 |
| **Better frailty status** |  |  |  |  |  |  |
| Pre-frail to not-frail | 0.24 | 0.27 | 0.32 | 0.26 | 0.30 | 0.34 |
| Frail to pre-frail | 0.25 | 0.25 | 0.24 | 0.35 | 0.34 | 0.35 |
| **Frailty transition to CVD** |  |  |  |  |  |  |
| Not-frail to CVD | 0.01 | 0.01 | 0.01 | 0.01 | 0.01 | 0.01 |
| Pre-frail to CVD | 0.01 | 0.01 | 0.01 | 0.01 | 0.01 | 0.01 |
| Frail to CVD | 0.01 | 0.01 | 0.01 | 0.01 | 0.01 | 0.01 |
| **Frailty transition to non-CVD death** |  |  |  |  |  |  |
| Not-frail to non-CVD death | 0.23 | 0.21 | 0.20 | 0.20 | 0.19 | 0.17 |
| Pre-frail to non-CVD death | 0.28 | 0.26 | 0.26 | 0.24 | 0.23 | 0.21 |
| Frail to non-CVD death | 0.37 | 0.35 | 0.36 | 0.33 | 0.31 | 0.27 |

**Supplemental Table S12.** Transition probability matrix between frailty states and to a CVD event (absorbing state), and to death from non-CVD causes (competing risk), according to area of residence, (i.e. major cities, inner regional and outer regional / remote of Australia) separately

|  | **FI** | | | **Fried** | |  |
| --- | --- | --- | --- | --- | --- | --- |
|  | Major cities  (n = 8290) | Inner Regional  (n = 5683) | Outer Regional / Remote  (n = 1851) | Major cities (n = 7377) | Inner Regional  (n = 5041) | Outer Regional / Remote  (n = 1653) |
| **1-year** |  |  |  |  |  |  |
| **Worse frailty status** |  |  |  |  |  |  |
| Not-frail to pre-frail | 0.19 | 0.20 | 0.20 | 0.24 | 0.24 | 0.22 |
| Pre-frail to frail | 0.13 | 0.13 | 0.14 | 0.07 | 0.08 | 0.08 |
| **Better frailty status** |  |  |  |  |  |  |
| Pre-frail to not-frail | 0.18 | 0.18 | 0.18 | 0.19 | 0.18 | 0.19 |
| Frail to pre-frail | 0.20 | 0.19 | 0.20 | 0.37 | 0.35 | 0.33 |
| **Frailty transition to CVD** |  |  |  |  |  |  |
| Not-frail to CVD | 0.01 | 0.01 | 0.01 | 0.01 | 0.01 | 0.01 |
| Pre-frail to CVD | 0.01 | 0.01 | 0.01 | 0.01 | 0.01 | 0.02 |
| Frail to CVD | 0.02 | 0.02 | 0.03 | 0.03 | 0.03 | 0.04 |
| **Frailty transition to non-CVD death** |  |  |  |  |  |  |
| Not-frail to non-CVD death | 0.01 | 0.01 | 0.01 | 0.01 | 0.01 | 0.01 |
| Pre-frail to non-CVD death | 0.02 | 0.02 | 0.02 | 0.01 | 0.01 | 0.02 |
| Frail to non-CVD death | 0.04 | 0.04 | 0.05 | 0.04 | 0.05 | 0.06 |
| **5-year** |  |  |  |  |  |  |
| **Worse frailty status** |  |  |  |  |  |  |
| Not-frail to pre-frail | 0.33 | 0.34 | 0.33 | 0.43 | 0.43 | 0.40 |
| Pre-frail to frail | 0.19 | 0.21 | 0.20 | 0.08 | 0.09 | 0.09 |
| **Better frailty status** |  |  |  |  |  |  |
| Pre-frail to not-frail | 0.33 | 0.30 | 0.30 | 0.34 | 0.33 | 0.34 |
| Frail to pre-frail | 0.30 | 0.29 | 0.29 | 0.43 | 0.41 | 0.37 |
| **Frailty transition to CVD** |  |  |  |  |  |  |
| Not-frail to CVD | 0.01 | 0.01 | 0.01 | 0.01 | 0.01 | 0.02 |
| Pre-frail to CVD | 0.01 | 0.01 | 0.01 | 0.01 | 0.02 | 0.02 |
| Frail to CVD | 0.01 | 0.01 | 0.01 | 0.02 | 0.02 | 0.02 |
| **Frailty transition to non-CVD death** |  |  |  |  |  |  |
| Not-frail to non-CVD death | 0.08 | 0.09 | 0.10 | 0.08 | 0.08 | 0.08 |
| Pre-frail to non-CVD death | 0.13 | 0.13 | 0.15 | 0.11 | 0.12 | 0.13 |
| Frail to non-CVD death | 0.22 | 0.22 | 0.25 | 0.19 | 0.21 | 0.25 |
| **10-year** |  |  |  |  |  |  |
| **Worse frailty status** |  |  |  |  |  |  |
| Not-frail to pre-frail | 0.30 | 0.30 | 0.30 | 0.40 | 0.39 | 0.37 |
| Pre-frail to frail | 0.16 | 0.17 | 0.16 | 0.06 | 0.07 | 0.07 |
| **Better frailty status** |  |  |  |  |  |  |
| Pre-frail to not-frail | 0.30 | 0.27 | 0.26 | 0.32 | 0.30 | 0.32 |
| Frail to pre-frail | 0.25 | 0.25 | 0.24 | 0.35 | 0.34 | 0.29 |
| **Frailty transition to CVD** |  |  |  |  |  |  |
| Not-frail to CVD | 0.01 | 0.01 | 0.01 | 0.01 | 0.01 | 0.01 |
| Pre-frail to CVD | 0.01 | 0.01 | 0.01 | 0.01 | 0.01 | 0.01 |
| Frail to CVD | 0.01 | 0.01 | 0.01 | 0.01 | 0.01 | 0.01 |
| **Frailty transition to non-CVD death** |  |  |  |  |  |  |
| Not-frail to non-CVD death | 0.20 | 0.22 | 0.23 | 0.18 | 0.19 | 0.21 |
| Pre-frail to non-CVD death | 0.26 | 0.27 | 0.29 | 0.22 | 0.23 | 0.26 |
| Frail to non-CVD death | 0.35 | 0.36 | 0.39 | 0.29 | 0.32 | 0.37 |

**Supplemental Table S13**. One-year, five-year and ten-year transition probability matrix from frailty state to CVD stratified by age-sex group

|  | **<74 years males** | **<74 years**  **females** | **>= 74 years males** | **>= 74 years females** |
| --- | --- | --- | --- | --- |
| **Frailty index** | **n = 4121** | **n = 5006** | **n = 3804** | **n = 5146** |
| **Transition probabilities after one year** |  |  |  |  |
| **Frail to CVD** | 0.03 | 0.01 | 0.05 | 0.03 |
| **Pre Frail to CVD** | 0.02 | 0.01 | 0.03 | 0.02 |
| **Not Frail to CVD** | 0.01 | 0.00 | 0.01 | 0.01 |
|  |  |  |  |  |
| **Transition probabilities after five years** |  |  |  |  |
| **Frail to CVD** | 0.10 | 0.05 | 0.18 | 0.11 |
| **Pre Frail to CVD** | 0.08 | 0.04 | 0.12 | 0.08 |
| **Not Frail to CVD** | 0.06 | 0.03 | 0.08 | 0.06 |
|  |  |  |  |  |
| **Transition probabilities after ten years** |  |  |  |  |
| **Frail to CVD** | 0.17 | 0.09 | 0.29 | 0.20 |
| **Pre Frail to CVD** | 0.14 | 0.07 | 0.23 | 0.16 |
| **Not Frail to CVD** | 0.12 | 0.06 | 0.19 | 0.13 |
|  |  |  |  |  |
| **Fried phenotype** | n = 3724 | n = 4532 | n = 3288 | n = 4376 |
| **Transition probabilities after one year** |  |  |  |  |
| **Frail to CVD** | 0.03 | 0.01 | 0.04 | 0.03 |
| **Pre Frail to CVD** | 0.01 | 0.01 | 0.02 | 0.01 |
| **Not Frail to CVD** | 0.01 | 0.00 | 0.01 | 0.01 |
|  |  |  |  |  |
| **Transition probabilities after five years** |  |  |  |  |
| **Frail to CVD** | 0.09 | 0.04 | 0.14 | 0.11 |
| **Pre Frail to CVD** | 0.07 | 0.03 | 0.10 | 0.07 |
| **Not Frail to CVD** | 0.06 | 0.03 | 0.08 | 0.06 |
|  |  |  |  |  |
| **Transition probabilities after ten years** |  |  |  |  |
| **Frail to CVD** | 0.15 | 0.07 | 0.23 | 0.17 |
| **Pre Frail to CVD** | 0.12 | 0.06 | 0.20 | 0.14 |
| **Not Frail to CVD** | 0.11 | 0.06 | 0.17 | 0.13 |


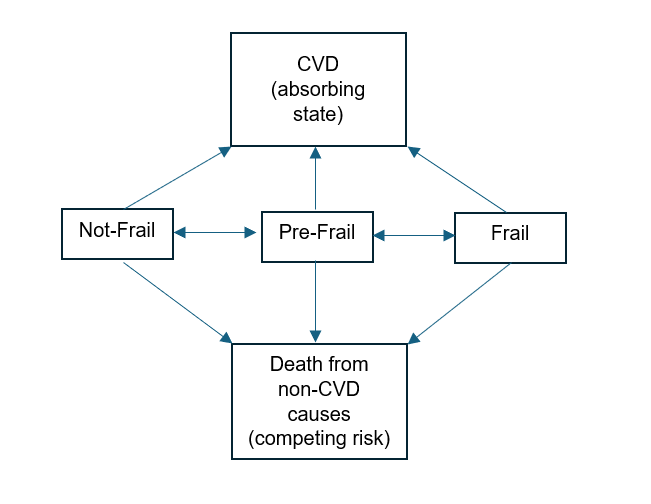


**Supplemental Figure S1**. State transition diagram incorporating death from non-CVD causes (competing risk) for sensitivity analyses


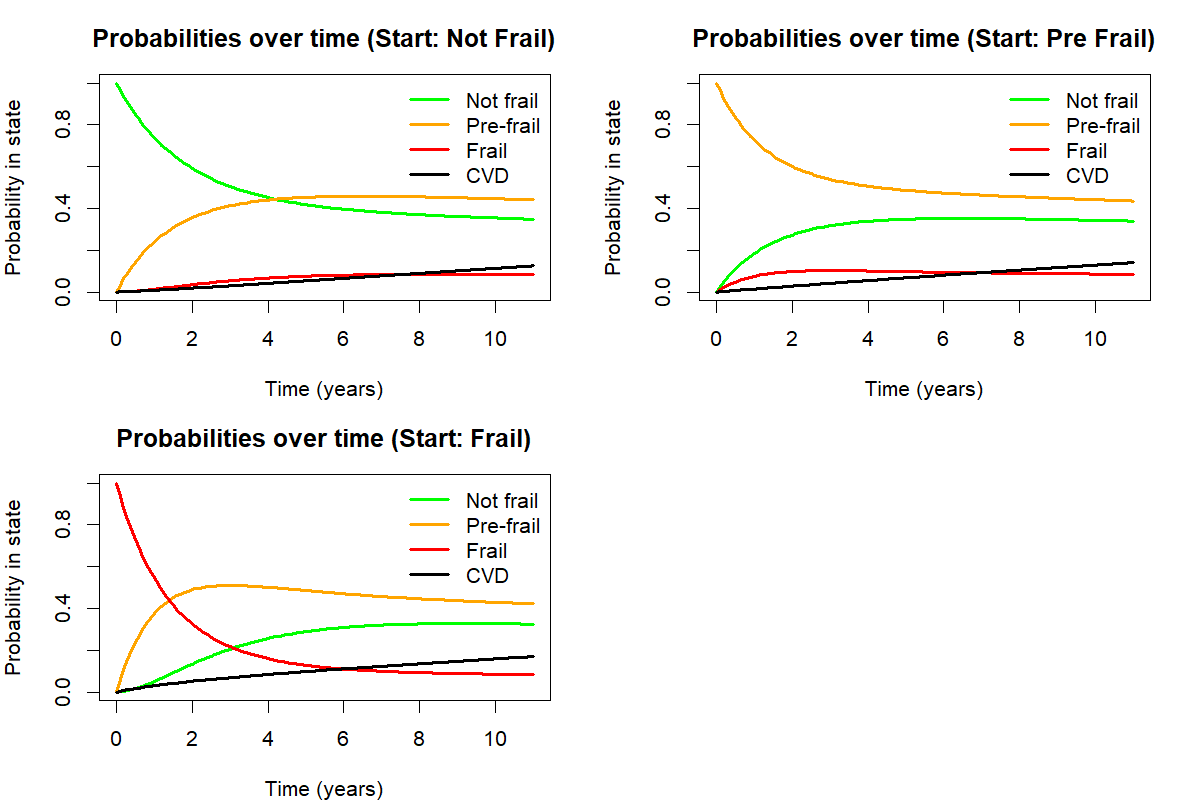


**Supplemental Figure S2.** Probabilities transitioning into different Fried phenotype frailty states and to a CVD event over time, starting from not-frail, pre-frail and frail
